# Degenerative Expansion of a Young Supergene

**DOI:** 10.1093/molbev/msy236

**Published:** 2018-12-21

**Authors:** Eckart Stolle, Rodrigo Pracana, Philip Howard, Carolina I Paris, Susan J Brown, Claudia Castillo-Carrillo, Stephen J Rossiter, Yannick Wurm

**Affiliations:** 1 School of Biological and Chemical Sciences, Queen Mary University of London, London, United Kingdom; 2 Departamento Ecología, Genética y Evolución, Facultad de Ciencias Exactas y Naturales, Universidad de Buenos Aires, Buenos Aires, Argentina; 3 Division of Biology, Kansas State University, Manhattan, Kansas; 4 Institut für Biologie, Martin-Luther-University Halle-Wittenberg, Hoher Weg 8, Halle, Germany

**Keywords:** social chromosome, accumulation of repetitive elements, evolution of polygyny, genome evolution, fire ants

## Abstract

Long-term suppression of recombination ultimately leads to gene loss, as demonstrated by the depauperate Y and W chromosomes of long-established pairs of XY and ZW chromosomes. The young social supergene of the *Solenopsis invicta* red fire ant provides a powerful system to examine the effects of suppressed recombination over a shorter timescale. The two variants of this supergene are carried by a pair of heteromorphic chromosomes, referred to as the social B and social b (SB and Sb) chromosomes. The Sb variant of this supergene changes colony social organization and has an inheritance pattern similar to a Y or W chromosome because it is unable to recombine. We used high-resolution optical mapping, k-mer distribution analysis, and quantification of repetitive elements on haploid ants carrying alternate variants of this young supergene region. We find that instead of shrinking, the Sb variant of the supergene has increased in length by more than 30%. Surprisingly, only a portion of this length increase is due to consistent increases in the frequency of particular classes of repetitive elements. Instead, haplotypes of this supergene variant differ dramatically in the amounts of other repetitive elements, indicating that the accumulation of repetitive elements is a heterogeneous and dynamic process. This is the first comprehensive demonstration of degenerative expansion in an animal and shows that it occurs through nonlinear processes during the early evolution of a region of suppressed recombination.

## Introduction

Recombination facilitates the removal of deleterious mutations and creates advantageous combinations of alleles. However, in some circumstances reduced recombination is favored. This occurs during the early evolution of supergenes, in which selection favors the suppression of recombination between haplotypes with advantageous combinations of alleles at different loci (Rice 1984; Wright et al. 2016). Because of interference among linked loci, reduced recombination also leads to reduced efficacy of selection, including reduced ability to remove deleterious mutations such as repeat insertions (Felsenstein 1974; Rizzon et al. 2002; Bachtrog 2006a; Dolgin et al. 2008; Dolgin and Charlesworth 2008). This phenomenon is strongest in supergene variants where recombination is fully suppressed, such as in sex chromosomes which harbor the supergene regions that have been studied the most. For example, because the Y (or W) chromosome does not occur in the homozygous state, genetic hitchhiking and background selection affect the entire length of its supergene region (Felsenstein 1974; Bachtrog 2006a; 2013; Wright et al. 2016). This results in the gradual degeneration of Y (and W) chromosomes, with two striking long-term effects: the loss of protein-coding genes and relative accumulation of repetitive elements (Charlesworth et al. 1994; Bachtrog 2006a), reducing gene density, and the length of the supergene region. This is particularly visible in the human Y chromosome which has approximately 14 times fewer genes and 5 times lower gene density, and is 2.7 times shorter than the X chromosome (Skaletsky et al. 2003; Ross et al. 2005).

The accumulation of repetitive elements is likely pervasive throughout low-recombination regions. For example, it is well-documented that centromeres, which generally have lower recombination rates, have higher repeat content than noncentromeric regions (Charlesworth et al. 1994). Accumulation of repeats can already happen at early stages of Y chromosome evolution as shown in *Drosophila miranda* (age 1.75 million years, i.e., ∼17.5 million generations) (Bachtrog et al. 2008; Krasovec et al. 2018) and *Silene latifolia* (age 11 million years, i.e., ∼7.3 million generations) (Krasovec et al. 2018). However in these sex chromosomes, more DNA has been lost overall than gained. Intriguingly, the supergene region of suppressed recombination on the hermaphrodite determining Y^h^ chromosome of papaya (7 million years old, i.e., ∼7 million generations) is approximately 2-fold larger than the homologous region in the X chromosome (Wang et al. 2012). Such findings of size increases in nonrecombining sex chromosomes suggest that large-scale accumulation of repetitive elements could precede gene loss (Hobza et al. 2017; Puterova et al. 2018). However, there are no convincing demonstrations of how or when such “degenerative expansion” occurs (Ming et al. 2007) in animals. This could be because repetitive regions are difficult to study, or because animal supergenes might transition rapidly to a phase of DNA loss and shrinkage. In contrast, some plant Y chromosomes appear to remain in the expansion phase for longer periods of time (Ming et al. 2007; Hobza et al. 2017). Furthermore, we know little about the relative roles of different types of DNA in degenerative expansion. The expansions of Y chromosomes in plants have been attributed to one or few repetitive elements (Hobza et al. 2006; Kubat et al. 2008; Kejnovský et al. 2013; Na et al. 2014), and analysis of platypus Y chromosomes suggests that multiple classes of repetitive elements may independently be amplifying in different Y chromosome lineages (Kortschak et al. 2009). Finally, other mechanisms such as segmental duplication might also be involved (Hobza et al. 2017).

We now know that supergene architectures are not rare and control variation in many complex ecological phenotypes (Schwander et al. 2014; Thompson and Jiggins 2014) thus increasing the importance of understanding the trade-offs involved in their evolution. The young social supergene system of the red fire ant *Solenopsis invicta* provides an ideal opportunity to examine the early effects of restricted recombination. The two variants of this supergene are carried by a pair of social chromosomes, referred to as the social B and social b (SB and Sb, respectively) chromosomes. This system controls a complex social phenotypic dimorphism where colonies have either one or up to dozens of reproductive queens (Keller and Ross 1998; Wang et al. 2013). The accumulation of unique SNP alleles indicates that recombination between the two variants has been suppressed for >350,000 years (i.e., >175,000 generations) over a chromosomal region encompassing >20 Mb and containing >400 protein-coding genes (Wang et al. 2013). The suppression of recombination in heterozygous individuals (i.e., individuals with the *Bb* genotype, with *B* marking the SB variant and *b* marking the Sb variant) has led to differentiation between SB and Sb throughout the entire length of the region (Pracana, Priyam, et al. 2017). SB can recombine in homozygote diploid *BB* queens. However, *bb* queens are never observed, either because they fail to reproduce, or because they die due to other intrinsic reasons (Gotzek and Ross 2007). Because Sb has no opportunity to recombine it should be affected by reduced efficacy of selection in a similar way to a Y or W chromosome.

To test whether degenerative expansion is an early effect of suppressed recombination, we apply a dual approach based on Bionano Genomics (BNG) Irys optical mapping and Illumina short-read sequence data.

## Results and Discussion

In a first step, we optically mapped one haploid fire ant male carrying the SB variant and one carrying the Sb variant (respectively referred to as the *B* and the *b* individuals). For each individual, we created a de novo assembly of optical contigs (supplementary information 1.1, Supplementary Material online), respectively amounting to 416 and 417 Mb total lengths (respective N50s of 1.58 and 1.41 Mb). We further assembled the *B* individual into optical chromosomes by combining optical contigs, genetic maps (Wang et al. 2013; Pracana, Priyam, et al. 2017) and reference sequence scaffolds (Wurm et al. 2011) (N50 of 22.60 Mb; supplementary information 1.2, Supplementary Material online).

### At Least Two Large Inversions between the Sb and SB Variants of the Social Chromosome

**Fig. 1.**
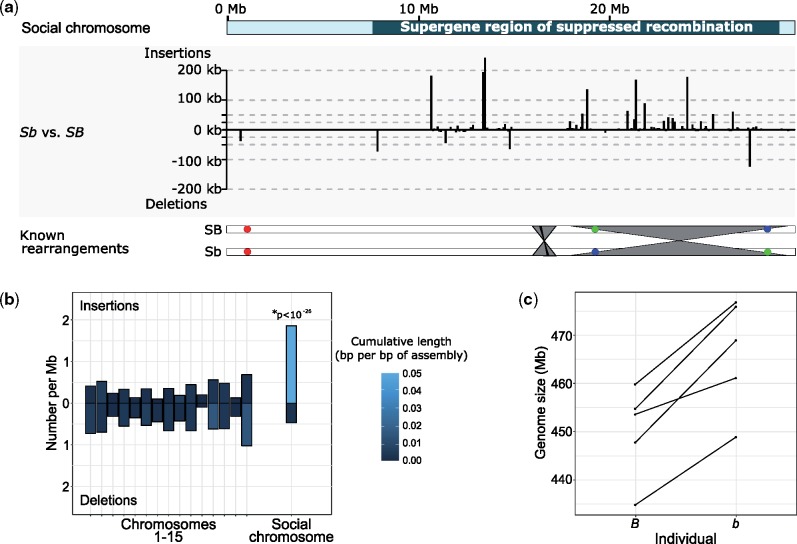
Accumulation of insertions in the *S. invicta* Sb supergene variant. (*a*) Graph: Distribution of insertions and deletions along the social chromosome are largely within the supergene region (located from position 7.7 to 28.6 Mb). Bottom: overview of known rearrangements between SB and Sb. Gray ribbons represent inversions detected in this study; black ribbon represents a previously known 48 kb inversion (within the gray ribbon); colored circles represent BAC-FISH markers A22, E17, E03 (Wang et al. 2013). (*b*) Frequency and cumulative length of insertions and deletions in the pairwise comparison of optical contigs between an *S. invicta b* and an *S. invicta B* individual. Insertions were not homogeneously distributed among chromosomes (χ^2^_d.f. = 15_ = 152, *P* < 10^−23^) with a significant enrichment exclusively on “social” chromosome 16, which carries the supergene region (*Z*-score = 11.1, Bonferroni-corrected *P* < 10^−26^). (*c*) Genome sizes estimated using k-mer frequency distributions from cleaned but unassembled Illumina sequence are higher in five *S. invicta b* individuals than in five paired *B* individuals from the native range of this species.

We first performed pairwise alignments between the optical assembly from the *b* individual and the optical chromosomes from the *B* individual to identify rearrangements characterizing the social chromosome supergene. We found two large-scale inversions between SB and Sb in *S. invicta.* The first spans approximately 10.5 Mb at the distal end of the social chromosome (fig. 1 , supplementary information 1.3, Supplementary Material online). The distal breakpoint colocates with the end of the supergene region as identified from the pattern of SB-Sb sequence differentiation (Wang et al. 2013; Pracana, Priyam, et al. 2017); this inversion likely represents a large inversion previously detected by fluorescence *in situ* hybridization (Wang et al. 2013). The second inversion is further upstream and spans 1.74 Mb (fig. 1, supplementary information 1.3, Supplementary Material online), colocating with a previously reported smaller (∼48 kb) inversion (Wang et al. 2013). The two rearrangements reported here between SB and Sb support the hypothesis that rearrangements inhibit potential double crossovers that would otherwise occur in the middle of a single large inverted region (Stevison et al. 2011). These two rearrangements are located in the second half of the supergene region, which suggests that additional undetected mechanisms or rearrangements suppress recombination in the first half of the supergene region (Wang et al. 2013; Pracana, Priyam, et al. 2017). The amount of neutral differentiation was similar between the two rearrangements (mean dS = 3.0×10^-3^ in the first inversion, dS = 2.5×10^-3^ in the second inversion; *t*-test, *P* = 0.47, supplementary information 1.3, Supplementary Material online). This suggests that, rather than representing different strata (Wright et al. 2014), the two inversions likely occurred in rapid succession, or that one or both appeared only after recombination had already been suppressed.

### Excess of Large Insertions in Sb in Comparison to SB

We performed pairwise alignments between the optical assemblies of the two individuals to identify large (≥3 kb) insertions and deletions (indels) (supplementary information 1.4, Supplementary Material online). The 187 deletions in the *b* individual were homogeneously distributed among the 16 chromosomes according to chromosome size (χ^2^_d.f.__ = __15_ = 24.02, *P* = 0.07). However, the social chromosome which carries the supergene region was significantly enriched in insertions (fig. 1*a* and *b*): this chromosome harbors 33.7% (55) of the 163 mapped insertions despite representing only 8.4% (29.61 Mb) of the superscaffolded genome (optical chromosomes: 350.94 Mb; χ^2^_d.f__. = __15_ = 152, *P* < 10^−23^). Similarly, the cumulative length of insertions on the social chromosome was 58.5% (1.43 Mb) of the cumulative length of all insertions (2.44 Mb), higher than would be expected if the insertions were homogeneously distributed across chromosomes. We then identified “overhangs,” unaligned regions that flank alignments between the optical assemblies of the *B* and the *b* individuals. Such overhangs either represent indels, highly divergent sequences, or are regions where an optical assembly is too fragmented for pairwise alignment to be successful. The cumulative amount of overhanging sequence indicates that the supergene region is 5.27 Mb larger in the *b* individual than in the *B* individual. This is a significantly greater difference than for chromosomes 1–15 (−1.43 to −0.25 Mb, χ^2^_d.f__. = __15_ = 83.25, Bonferroni-corrected *P* < 10^-14^). Combining the indels detected with both methods, the *b* variant of the supergene region is 31.7% longer (total length 27.52 Mb) than the *B* variant (20.9 Mb). Importantly, due to higher contiguity of the assembly from the *B* individual than the assembly from the *b* individual, a bias in power would be toward detecting excess sequence in SB rather than Sb (cf. supplementary information 1.2 and 1.4, Supplementary Material online). Thus the 31.7% increase of length of Sb is likely to be an underestimate.

### K-mer Distribution Analysis Show That *b* Individuals Have Larger Genomes than *B* Individuals

To corroborate our results, we obtained Illumina short-read sequence data for five pairs of ants, each containing one *B* male and one *b* male taken from either the same colony (three pairs) or neighboring colonies of ≤50 m apart (two pairs). All pairs were taken from one of three locations spanning the native South American range of *S. invicta.* We independently estimated genome size and the proportion of repetitive sequence in the genome of each sample using the distribution of 21-nucleotide k-mer sequences (Sun et al. 2018) (supplementary information 1.5, Supplementary Material online). Estimated genome sizes for *b* samples were 3.59% larger (95% confidence interval: 2.02–5.16%) than those of *B* samples (paired one-sided *t*-test: *P* < 0.002; fig. 1*c*). Using a previous estimate that the SB supergene region represents 4.5% of the genome (Pracana, Priyam, et al. 2017), and assuming that the difference in genome size between the *b* and *B* samples is entirely due to the increase in size of Sb in the supergene region, these data indicate that the Sb variant of the supergene is 79.8% (44.9–114.7%) larger than the SB variant. These results are consistent with the optical-mapping-based inference that Sb is at least 31.7% larger than SB; furthermore they are unbiased because these results were determined without a reference genome. The variation between pairs is likely due to differences in repeat content and hence genome size, consistent with independent quantifications of repeat frequencies (see below).

### *S. invicta b* Individuals Have Higher Repeat Content than *B* Individuals, But the Repetitive Elements Responsible for This Differ between Pairs of Individuals

Using the same five pairs of individuals as used for the k-mer analysis, we comprehensively quantified repetitive elements (repeats) using reference-free repeat assembly from short reads (Goubert et al. 2015). We found that *b* individuals contain 2.23% (range: 1.46–3.02%), that is, 10.02 Mb (range: 6.57–13.58 Mb) more repeats than *B* individuals (paired one-sided *t*-test: *P* < 0.0009, supplementary information 1.6, supplementary table S8, Supplementary Material online). K-mer analysis shows qualitatively similar results (supplementary information 1.5, Supplementary Material online). These results are consistent with the Sb supergene variant being 47.94% larger than the SB variant, again in line with the idea that the 31.7% difference in size observed in optical maps is an underestimate.

Interestingly, the general increase in size of the Sb supergene variant was not due to one or few types of repeats (supplementary information 1.6, supplementary fig. S9, Supplementary Material online). We found a consistent increase in the number of repeats from 14 superfamilies, but their cumulative length accounted for only 3.09 Mb of the observed average increase of 10.02 Mb (supplementary table S8, Supplementary Material online). The genomic content that accounted for the additional increase was split between other repeat superfamilies in a manner that varied between pairs of individuals. For example, there were even 1.68 Mb fewer centromeric satellite repeats in one *b* than its paired *B* individual, whereas in the other four pairs, the *b* individual had 1.65–10.48 Mb more such repeats (supplementary information 1.6, supplementary table S8, Supplementary Material online). This indicates that degenerative expansion occurred in the lineages of each of the *b* variants we sampled, but that different repeats have increased in prevalence in these different lineages. Alternatively, degenerative expansion may be a dynamic process whereby repeats regularly expand and such expansions are regularly lost again. Furthermore, we find consistently increased prevalence of some nonautonomous DNA elements and variable degrees of increase of satellites in *b* individuals, indicating that expansive degeneration is not due to autonomous repeat elements alone. Instead, in line with documented variation in repeat content across platypus Y chromosomes (Kortschak et al. 2009), our results suggest that other mechanisms such as segmental duplication of repeat-rich genomic regions (Ishizaki 2002) also contribute to degenerative expansion.

### Supergene Inversions and Insertion Accumulation Are Consistent across Three Fire Ant Species

Several close relatives of *S. invicta* are also socially polymorphic. In these species, social polymorphism is associated with the *Gp-9* locus that marks the social supergene in *S. invicta* (Krieger and Ross 2005) although it is not currently known whether they also carry the supergene. Therefore, to test whether these species indeed carry the supergene and the same *B-b* differences in chromosomal structure and repeat content, we created optical assemblies for one *Gp-9 B* sample and one *Gp-9 b* sample from each of the two congeners *S. quinquecuspis* and *S. richteri* (supplementary information 1.1, Supplementary Material online). We identified large indels between the *B* and *b* optical assemblies in both of these species (supplementary information 1.4, Supplementary Material online), and performed phylogenetic analyses based on presence and absence of indels present in at least two individuals. We found that for each of chromosomes 1–15, individuals clustered by species. In contrast, in a tree built using the supergene region of the social chromosome, the *b* individuals clustered separately from the *B* individuals, similarly to what was previously shown for the *Gp-9* locus (Krieger and Ross 2002). These data demonstrate that the supergene region exists in all three species, and that it likely has a single origin. These conclusions are further corroborated by inversions shared across species (supplementary information 1.3, Supplementary Material online). The optical assemblies of the related species had lower contiguity than for *S. invicta* but provided the power to compare distributions of insertions and deletions. In both additional species, the supergene region in the *b* sample had a highly significant enrichment of insertions but not deletions in comparison to the *B* sample and to the rest of the genome (supplementary information 1.4, Supplementary Material online), consistent with degenerative expansion being a pervasive feature of Sb.

Our phylogenetic analysis of the indels in the supergene suggest that the supergene either originated in the common ancestor of the three species or that it arose more recently and spread between lineages by introgressive hybridization. To discriminate between the two hypotheses, we dated the split between the three species based on full mitochondrion sequences (supplementary information 1.8, Supplementary Material online). The mitochondrial phylogenetic tree topology is consistent with previous inferences (Gotzek et al. 2010) estimating common ancestry between *S. quinquecuspis*, *S. richteri,* and *S. invicta* to approximately 367,000 years ago (0.25–0.50 million years, supplementary information 1.8, supplementary fig. S12, Supplementary Material online). This estimate is similar to the age estimate for the social chromosome (0.35–0.43 million years) (Wang et al. 2013) suggesting similar ages of the social chromosomes and the node containing the socially polymorphic fire ant species. This and previous phylogenetic analyses (Krieger and Ross 2002) support the idea that the social chromosome evolved in the common ancestor of these species. However, one *S. invicta* sequence obtained from NCBI (HQ215540) showed a paraphyletic relationship with respect to its putative conspecifics, with a much earlier divergence based on mitochondrial sequences (supplementary information 1.8, Supplementary Material online). If the species identity of this individual is correct, then the evolutionary history of the social chromosome may be more complex, potentially involving introgression of the social chromosome across hybridizing species (Jay et al. 2018).

### The Causes of Degenerative Expansion

In summary, Sb contains at least 30%, but likely 48–80% more DNA content than SB. Previous work has described only few differences in content of protein-coding genes between Sb and SB (Wang et al. 2013; Pracana, Levantis, et al. 2017). Our results thus suggest that Sb is undergoing degenerative expansion in three *Solenopsis* species. But what causes this increase in chromosome size?

In nonrecombining chromosomes, background selection (Kaiser and Charlesworth 2010) and genetic hitchhiking (Bachtrog 2004) both cause a reduction on the effectiveness of purifying selection (Bachtrog 2008). It has been shown that the fixation rate of an allele in a nonrecombining chromosome is dependent on how deleterious the allele is, with highly deleterious mutations having a lower fixation rate than mutations that are less deleterious (Kaiser and Charlesworth 2010). Given the relative rarity of large indel polymorphisms outside the supergene region, and the low frequencies of large indels in other species (Sudmant et al. 2015; Long et al. 2018), we can assume that large insertions and deletions generally have a higher fitness cost than point mutations. Deletions are generally thought to be more deleterious than insertions because they involve the complete removal of genetic information, an assumption that is supported by the lower frequency of standing variation in deletions than insertions in human populations (Sudmant et al. 2015). If insertions are similarly less deleterious than deletions in the social chromosome system, insertions would become fixed at a higher rate, which would thus explain the increase in the chromosome size of Sb. To illustrate this process, we performed forward simulations of populations of individuals carrying a single nonrecombining haploid chromosome (supplementary information 1.10, Supplementary Material online). Under conditions where deletions have a higher fitness cost than insertions, the simulations indeed show an average increase in the size of chromosomes over time (supplementary fig. S13, Supplementary Material online).

Unlike previous findings that within a species, specific repeat classes including (micro-) satellite repeats (Hobza et al. 2006; Kubat et al. 2008; Shanks et al. 2008; Kejnovský et al. 2013) or retrotransposons (Na et al. 2014), we unexpectedly observed that the largest repeat expansions are different in each Sb chromosome we studied. A possible interpretation is that we sampled independent Sb lineages, each undergoing the same process of degenerative expansion separately, since otherwise we would expect all individuals to carry similar haplotypes (Kaiser and Charlesworth 2009). Population subdivision is expected to accelerate the fixation of deleterious mutations, as it reduces the effective size of each population (Combadão et al. 2007).

The fire ant supergene system is different from many sex chromosome systems in that male ants are haploid. The presence of such an important haploid stage can have a major effect on evolutionary dynamics because alleles that would be recessive in a diploid individual are instead completely exposed to selection. As a consequence, one could expect that the purging effects of purifying selection would be stronger in the Sb variant of the fire ant supergene than in supergenes in diploid systems. This scenario is supported by studies of plant and algal species with important haploid stages (Chibalina and Filatov 2011; Arunkumar et al. 2013; Lipinska et al. 2017; Coelho et al. 2018; Immler and Otto 2018; Sandler et al. 2018). However, simulations performed in other studies have shown that the increased exposure of alleles to selection in haploids can also increase the strength of background selection and, therefore, the fixation rate of deleterious mutations (Engelstädter 2008; Kaiser and Charlesworth 2010). Consequently, the effects of the haploid life stage may result from the balance between increased purifying selection and increased background selection. Strong selection against deletions in haploid males would prevent their fixation in a population, whereas strong background selection would contribute to the accumulation and fixation of insertions. Such a dynamic would lead to the type of rapid accumulation of repeats we see in Sb.

### A Brake against Degenerative Expansion

Nonrecombining chromosomes are not expected to increase in size indefinitely. As the chromosome degenerates, the number of intact functional elements in the nonrecombining region decreases, removing the fitness cost of most mutations (Kaiser and Charlesworth 2010), including deletions. Ultimately, if insertions and deletions occur at a similar rate, they would drift in the population, and the chromosome would cease to grow (illustrated by simulations in supplementary fig. S14, Supplementary Material online). The process of degeneration is thought to be accelerated by mechanisms of dosage compensation (Charlesworth 1978; Mank 2013; Wright et al. 2016) and gene relocation (Bachtrog 2006a; Ming et al. 2007; Hobza et al. 2017; Lipinska et al. 2017), which transfer functional elements from the nonrecombining chromosome to its pair or to other chromosomes. Accordingly, empirical studies have shown that loss of expression precedes gene loss (Bull 1978; Campbell 1982; Joseph and Kirkpatrick 2004; Chibalina and Filatov 2011; Beaudry et al. 2017; Crowson et al. 2017; Coelho et al. 2018; Immler and Otto 2018; Sandler et al. 2018); thus genes that have no expression in the haploid life stage are more likely to be lost. Most studies of gene expression in the fire ant system (Wang et al. 2008; Nipitwattanaphon et al. 2013; Wang et al. 2013; Pracana, Levantis, et al. 2017) have relied on microarrays that include only a subset of the protein-coding genes in the genome (Wang et al. 2007). Nevertheless, these studies suggest that the supergene region includes a large proportion of the genes with differential expression between individuals with alternative SB/Sb genotypes in queens, workers and males, implying that SB and Sb may differ at regulatory sites (Wang et al. 2008; Nipitwattanaphon et al. 2013; Wang et al. 2013; Pracana, Levantis, et al. 2017). However, there is no evidence of either large-scale gene expression loss in Sb or of the accumulation of a large number of loss-of-function mutations (Wang et al. 2013; Pracana, Priyam, et al. 2017). Furthermore, the vast majority of *S. invicta* genes expressed in females are also expressed in haploid males (Nipitwattanaphon et al. 2014) and thus, as discussed above, likely to be under strong purifying selection. Lastly, an analysis of RNAseq data from SB/Sb queens found no evidence of systematically higher (or lower) expression of alleles on SB compared with those on Sb (Wang et al. 2013). This contrasts with results from *Drosophila miranda* neo-sex chromosomes where higher expression on the neo-X chromosome relative to the neo-Y is seen as evidence of degeneration of the neo-Y (Bachtrog 2006b). To summarize, the Sb supergene variant is likely in a stage where degenerative expansion is still ongoing.

Interestingly, many Y (and W) chromosomes in late stages of evolution have greatly decreased in size relative to the X (and Z) chromosomes. A possible explanation for this is the occurrence of rare large deletions, which can become fixed in the population as long as they do not encompass the last remaining functional loci of the chromosome. Large deletions are known to be caused by ectopic recombination between homologous repeats in distant positions in the chromosome (Devos et al. 2002; Roehl et al. 2010). An excess of such deletions, in comparisons to insertion processes, would therefore lead to a decrease in the size of the Y chromosome over time (illustrated by the simulations presented in supplementary fig. S15, Supplementary Material online).

## Conclusion

Our findings from ants add to a growing body of evidence that nonrecombining chromosomes can increase in size through degenerative expansion. As reported for some plants and algae, such expansions may be accelerated by a haploid life stage, although they might also occur more widely. For example, in stickleback fish, a nascent Y chromosome that is cytologically indistinguishable from the X chromosome includes Y-specific insertions and duplications (Peichel et al. 2004). More recent findings from *Drosophila miranda* also support a nearly 3-fold expansion in the neo-Y chromosome via the accumulation of repeat sequences (Mahajan et al. 2018). Similarly, the older *Drosophila hydei* Y chromosome is smaller than its X chromosome counterpart, but carries some of the largest introns of the genome (≥3.6 Mb) (Reugels et al. 2000), perhaps a remnant of past chromosome-wide expansion. More work is now needed to determine whether such expansions have occurred more widely across the tree of life, as well as to resolve the underlying mutational processes.

## Materials and Methods

### Ant Collections

We collected *Solenopsis invicta*, *Solenopsis quinquecuspis,* and *Solenopsis richteri* fire ants from their native South American range, identified species using partial sequencing of the mitochondrially encoded cytochrome c oxidase I gene and confirmed colony social form using on a *Gp-9* marker assay (Krieger and Ross 2002) (supplementary information 1.1 and 1.8, Supplementary Material online). For K-mer-based analyses, we selected five pairs of one *B* individual and one *b* individual, each pair originating from the same colony (*n* = 3, from two geographic locations with a distance of >2,000 km), or from two neighboring colonies (*n* = 2, colony distance 5 and 50 m, both from the same geographic location and approximately 200 and >2,000 km from the other two locations; supplementary information 1.1 and 1.8, Supplementary Material online).

### Optical Mapping

For each of the of the three *Solenopsis* species, we extracted high-molecular weight (HMW) DNA from one haploid male pupae carrying the *B* genotype at the *Gp-9* locus and one carrying the *b* genotype following the BNG IrysPrep animal tissue protocol (supplementary information 1.1, Supplementary Material online). Each sample was optically mapped using BNG nanochannel arrays for 30 cycles providing ∼130-Gb sequence data in molecules ≥100 kb (range 71–203 Gb; i.e., on average 290-fold genome coverage). These raw BNG Irys optical molecules were processed, analyzed, and *de novo* assembled in IrysView (BNG, v2.4, scripts v5134, tools v5122AVX; supplementary information 1.1, Supplementary Material online).

### Optical Assembly Comparisons, Optical Chromosomes

Comparisons between optical assemblies were performed by pairwise alignments using BNG IrysView (v2.4; supplementary information 1.3, 1.4, and 1.9, Supplementary Material online). Large (≥3 kb) insertions and deletions (indels) were detected as described previously (Kawakatsu et al. 2016). A reciprocal alignment between *S. invicta* optical assemblies (*b* and *B*) yielded nearly identical results (95% of indel sites were recovered; data not shown), indicating high consistency of indel detection. We placed and oriented the optical contigs of the *S. invicta B* optical assembly onto the 16 linkage groups in the *S. invicta* genetic map (Pracana, Priyam, et al. 2017) using the alignment between the optical contigs and the scaffolds of the *S. invicta B* reference genome assembly (Wurm et al. 2011) (GCF_000188075.1; supplementary information 1.2, Supplementary Material online). The small portion of ambiguous placements of the optical contigs from this individual were resolved using information from optical contigs of the additional males.

### Phylogenetic Analysis

For phylogenetic tree reconstruction and dating, we used mitochondrial sequences generated from Illumina short-read data (supplementary information 1.7 and 1.8, Supplementary Material online). We additionally inferred phylogenetic relationships between samples based on presence and absence of shared indels detected in pairwise comparisons in at least two individuals in regions that had information (coverage) in all six individuals.

## Data Availability

The data sets generated and analyzed during the current study are available from NCBI (BioProject PRJNA397545: SUPPF_0000001241—SUPPF_0000001246, and BioProject PRJNA396161) and Genbank (accessions MF592128—MF592133). Hybrid assembly (BionanoGenomics) files, draft reference sequence assembly improvements (AGP), and optical assemblies (BionanoGenomics cmaps) can be downloaded from https://wurmlab.github.io/data/optical_mapping, last accessed January 26, 2019.

### Computer Code

Further details for specific analyses can be found in the supplementary information 1, Supplementary Material online. Bionano analysis scripts are available at https://github.com/estolle/BioNano-Irys-tools, and the simulation code is at https://github.com/wurmlab/simulate_chromosome_length_evolution, last accessed January 26, 2019.

## Supplementary Material

Supplementary data are available at *Molecular Biology and Evolution* online.

## Supplementary Material

Supplementary Data
