# Supplementary material for: Degenerative Expansion of a Young Supergene
Source: Mol Biol Evol. 2018 Dec 21;36(3):553–61. doi: 10.1093/molbev/msy236 (PMC6389315; doi:10.1093/molbev/msy236)
Supplement: Supplementary Data [file msy236_supp.zip › Suppl.2.pdf]

Degenerative expansion of a young supergene

Supplementary Information

Eckart Stolle, Rodrigo Pracana, Philip Howard, Carolina I. Paris, Susan J. Brown, Claudia Castillo-Carrillo, Stephen J. Rossiter, Yannick Wurm

Suppl. Information 1.1. Creation of optical assemblies for social chromosome variants from three fire ant species 3
Samples 3
Irys optical mapping of six samples 3
De novo assembly of optical contigs from each sample 4
Suppl. Information 1.2. Optical chromosome assembly for *Solenopsis invicta* 6
Creation of a “core” optical reference assembly for the *S. invicta B* sample 6
Optical mapping of the fire ant genome substantially increases reference genome contiguity 6
Detecting and resolving ambiguities and inconsistencies in the reference genome 7
Suppl. Information 1.3. Large scale rearrangements detected by comparing optical assemblies 8
Rearrangements between SB and Sb in the three species 8
Additional rearrangements and inversions in the genomes of the three fire ant species 11
Suppl. Information 1.4. Detection of insertions and deletions from optical mapping data 14
Insertions and deletions flanked on both sides by aligning sequence 14
“Overhanging” insertions and deletions between *B* and *b* 19
Total change in chromosome sizes in the *b* individual 21
Suppl. Information 1.5. K-mer based estimation of genome sizes of 10 haploid males 22
Suppl. Information 1.6. Comparison of repeat content between individuals carrying alternate variants of the supergene 28
Repeats detected from short paired-end reads 28
Large tandem repeats detected from the optical assemblies 31
Suppl. Information 1.7. Neighbor-Joining trees for each chromosome, based on shared insertion/deletion polymorphism 33
Suppl. Information 1.8. Phylogeny and divergence time estimation 38
Species identity 38
Divergence time estimation based on full mitochondrial sequences 39
Suppl. Information 1.9. Optical map alignment parameters 43
Suppl. Information 1.10. Simulating degenerative expansion 43
Other supplementary files in Supplementary Material online 47
References 47

Suppl. Information 1.1. Creation of optical assemblies for social chromosome variants from three fire ant species

Samples

For optical mapping, male pupae were collected from colonies in Brazil (*Solenopsis invicta* -20.13, -56.69) and Argentina (*Solenopsis quinquecuspis* -33.44, -59.07; *Solenopsis richteri* -32.74, -61.82) and immediately flash-frozen in liquid Nitrogen (permits: Brazil: Maria Cristina Arias (U Sao Paulo), permit number for exporting biological material: 14BR015531/DF; Argentina: Carolina Paris, Eckart Stolle, Entre Ríos collection permit 007/15 and federal exportation permit 282/2016; Santa Fé collection permit 433/02101-0014449-4 and federal exportation permit 25253/16). In addition, for resequencing we sampled *S. invicta* male pupae and adults from colonies in Brazil (four colonies -20.48, -54.75; two colonies -20.13, -56.69, one colony -29.92; -51.76, permit #14BR015531/DF to Prof. Maria Cristina Arias, U Sao Paulo). An overview of samples used and their genotypes is in Suppl. Table S9. To determine the species of each sample, we sequenced the mitochondrially encoded cytochrome c oxidase I (COI) locus of each sample and determined its position in the *Solenopsis* phylogeny. This approach used for this is detailed in Suppl. Information 1.8.

In *Solenopsis invicta*, the *B* and *b* alleles of the *Gp-9* gene are markers of the SB and the Sb supergene variant, respectively (Krieger and Ross 2002; Shoemaker and Ascunce 2010; Wang et al. 2013); this marker is also associated with social form in other socially polymorphic fire ant species (Ross et al. 2003). For each of the three species *Solenopsis invicta, Solenopsis quinquecuspis* and *Solenopsis richteri*, we determined the variant of the social chromosome based on a *Gp-9* marker assay (Krieger and Ross 2002) from either total Phenol-Chloroform extracted DNA (Hunt and Page 1995) or using a leg and antenna following the single-fly-prep protocol (Gloor et al. 1993).

Irys optical mapping of six samples

For each sample, we extracted high molecular weight DNA from a single flash-frozen fire ant male pupa following the Bionano Genomics (BNG) Irys prep animal tissue protocol (IrysPrep Animal Tissue - Dounce Tech Note v1.1.12). In brief, a single fire ant pupa was homogenized in a 7 mL dounce grinder containing 1 mL homogenization buffer (10 mM Tris, 10 mM EDTA, 100 mM NaCl, pH 9.4). The homogenate was embedded in low melt agarose plugs (Bio-Rad). The plugs were washed overnight at 50°C for at least 16 hours in 5 mL homogenization buffer and 200 µL 600 mAU/ml proteinase K (Qiagen Puregene). The following day the wash was repeated with fresh buffer and proteinase K for 2 hours at 50°C before adding 50 µL 100 mg/mL; 7000 units/mL, RNaseA solution and incubating for one hour at 37°C. The plugs were then washed at room temperature for 15 minutes 4 times in 5 mL homogenization buffer, repeated 5 times in 5 mL TE buffer (10 mM Tris, 1 mM EDTA). Then the plugs were removed with a spatula, blotted dry on a KimWipe (Kimberly-Clark) and transferred to a 1.5 mL microfuge tube where 2 µL of 0.2 U/µL Gelase (Cambio) was added for digestion at 43°C for 45 minutes. The solution was then pipetted onto a 0.1 µm dialysis membrane (Millipore) floating on 7 mL TE in a petri

dish. The resulting pure DNA sample was then pipetted from the filter to a new microfuge tube and left at room temperature to equilibrate at least overnight.

Following quantification (Qubit, Thermo Fisher), 300 ng of ultra-HMW DNA was nicked (1 µL 10 U/µL Nt.BspQI for 2 hours at 37°C), labelled (1.5 µL 10x fluorescent labelling mix, BNG, 60 min at 72°C), repaired (0.4 µL 50x BNG repair mix, 1 µL 40 U/µL Taq DNA ligase, NEB, 30 min at 37°C) and stained (1.5 µL BNG DNA stain containing yoyo-1, overnight) following the BNG IrysPrep Reagent Kit protocol. The labelled DNA was then applied to BNG nanochannel arrays on a BNG Irys genome mapping system for 30 cycles.

De novo assembly of optical contigs from each sample

We performed *de novo* assembly of the optical data from each of the six sequenced individuals. Raw BNG Irys optical molecules were processed, analyzed and assembled in IrysView (BNG, v2.4, scripts v5134, tools v5122AVX). All molecules shorter than 100 kb were excluded prior to analysis. For each sample, we proceeded in a two-step process. We first determined dataset noise parameters with the Molecule Quality Report based on comparison to the *S. invicta* draft sequence assembly (GCF_000188075.1 from NCBI; (Wurm et al. 2011)). We used these noise parameters to perform an initial *de novo* assembly (settings: BNG medium, minimum molecule length of 150 kb). We used this initial assembly to again perform a noise parameter assessment of raw optical reads using Molecule Quality Report. Using the resulting revised noise parameters, we performed a final assembly (settings: BNG medium, minimum molecule length of 100 kb).

The resulting assemblies for the *S. invicta* SB and Sb and the *S. quinquecuspis* SB samples were of comparable quality (Suppl. Table 1) and the assembly of *S. quinquecuspis* Sb more fragmented. In contrast, the assemblies for the *S. richteri* samples had lower contiguities, likely because optical reads from these samples were shorter than for the other samples. Optical assemblies (cmaps) are available for download from https://wurmlab.github.io/data/optical_mapping.

Suppl. Table S1. Optical assembly statistics.

	S. invicta		*S. quinquecuspis*		*S. richteri*		*S. invicta*
	**SB**	**Sb**	**SB**	**Sb**	**SB**	**Sb**	**SB optical chromosomes**
**Raw data**	161 Gb	136 Gb	90 Gb	122 Gb	71 Gb	203 Gb	n/a
**Raw data N50**	167 kb	186 kb	239 kb	223 kb	193 kb	138 kb	
**Number of optical contigs**	366	409	310	616	799	907	123
**Total length**	416 Mb	417 Mb	419 Mb	426 Mb	400 Mb	353 Mb	420 Mb
**N50**	1.58 Mb	1.41 Mb	2.10 Mb	0.89 Mb	0.61 Mb	0.44 Mb	22.60 Mb
**Coverage**	82×	80×	83×	74×	69×	55×	
**Optical contigs ≥2 Mb (number)**	52	43	67	14	0	0	
**Optical contigs ≥2 Mb (total length)**	152 Mb	129 Mb	219 Mb	36 Mb	0 Mb	0 Mb	
**Optical contigs ≥2 Mb (% of total assembly length)**	36%	31%	52%	8%	0%	0%	

Suppl. Information 1.2. Optical chromosome assembly for *Solenopsis invicta*

Creation of a “core” optical reference assembly for the *S. invicta B* sample

We created a reference optical assembly to determine the chromosomal positions of the optical contigs in our assemblies. For this, we used the optical assembly produced from the *S. invicta B* male because the reference genome assembly for this species is also based on a *B* male (GCF_000188075.1) (Wurm et al. 2011) and no reference genome assembly has been produced for the other two species. 249 Mb (69.9%) of the reference genome assembly has previously been placed into 16 chromosomal linkage groups using a consensus between seven genetic maps (Pracana et al. 2017).

We aligned the optical contigs of the *S. invicta B* male to the reference genome assembly using BNG IrysView (v2.4) using alignment settings as outlined in Suppl. Information 1.9. There were 219 reference scaffolds that matched optical contigs, which we used to place and orient 236 optical contigs (N50: 1.66 Mb; total length: 319 Mb). Optical mapping data of the *b* male provided unambiguous evidence for the further placement of 23 previously unplaced optical contigs. The resulting assembly included 259 optical contigs that were placed into 16 linkage groups (“optical chromosomes”, 350.94 Mb) as well as 107 unplaced optical contigs, with a total length of 419 Mb and a N50 of 22.60 Mb. This optical chromosome assembly provided a straightforward representation of genome architecture (Schwartz et al. 1993) and was then used as an optical reference for directly mapping optical contigs and thus detect chromosome-scale rearrangements. The *S. invicta* optical chromosomes as well as assignments of each species’ cmaps to chromosomes can be downloaded from https://wurmlab.github.io/data/optical_mapping.

Optical mapping of the fire ant genome substantially increases reference genome contiguity

To generate a high quality reference genome assembly for *S. invicta,* we aimed to superscaffold the current highly fragmented reference sequence assembly (Wurm et al. 2011) (69,511 contigs and scaffolds; total length: 396 Mb; N50: 0.56 Mb). For this, we used a hybrid assembly approach with the optical assembly obtained for the *S. invicta B* sample (above). Hybrid assembly aligned *BspQ*I sites in the optical contigs to those in the reference genome, and resulted in 146 optical contigs being used to superscaffold 158 of the reference scaffolds into 93 hybrid scaffolds (total length: 213 Mb; N50: 3.30 Mb, further data (hybrid assembly (BionanoGenomics) files available to download from https://wurmlab.github.io/data/optical_mapping). The N50 of the reference genome was thus improved by 160% (N50: 1.46 Mb; 69,446 contigs and scaffolds; total length: 464 Mb). The total length of 464 Mb of this hybrid assembly is substantially larger than the optical assembly of 420 Mb (350.94 Mb optical chromosomes and additional unplaced optical contigs). This discrepancy highlights the main limitation of this approach: only 678 of the reference genome scaffolds (287 Mb) contained sufficient (*i.e.*, at least five) *BspQ*I sites for comparison with optical contigs. In

practice, this meant that scaffolds shorter than approximately 100 kb could not be compared to optical contigs in this species and therefore were not placed and assembled. This leads to an inflation of the overall assembly size because gaps where such contigs were not placed are filled with “N”s and at the same time the contigs remain in the assembly as unplaced.

Detecting and resolving ambiguities and inconsistencies in the reference genome

To identify potential errors in the reference genome assembly, we performed extensive comparisons of the improved reference assembly, the optical assembly, and the genetic map. This integrated approach resolved the ambiguous placement of 31 reference genome scaffolds: we oriented 20 scaffolds, corrected the orientation of 3 scaffolds and placed 14 previously unmapped scaffolds.

Additionally, we detected two main types of sequence-level inconsistencies between the optical contigs and the reference genome. There were 9 cases of the first type, in which different portions of an optical contig aligned to multiple scaffolds from different chromosomes or from distant positions within the same chromosome. In all cases, information from the genetic map was consistent with the optical contigs. These inconsistencies thus likely reflect situations where sequences from distinct locations in the genome were incorrectly assembled into the same scaffold in the reference genome assembly. A corrected version of the GCF_000188075.1 reference assembly and a file with details of corrections and scaffold order is given as AGP file available for download from https://wurmlab.github.io/data/optical_mapping.

In the second type of inconsistency, one assembly included regions missing from the other (example in Suppl. Fig. S1A). We found 47 regions of >3kb present in the reference genome but missing from the optical assembly, representing 0.38 Mb in total (Suppl. Fig. S1B, Suppl. Information 1.2). The molecular biology steps involved in optical sequencing include no amplification or other steps that could lead to consistent skipping of certain DNA fragments. In consequence, the majority of these sequences likely represent true structural differences between the individual used for the reference genome and the individual used for the optical assembly. We similarly found 615 regions of >3kb that are present in the optical assembly but missing from the reference genome, representing 4.38 Mb in total (Suppl. Fig. S1B). While some of these may also represent biological differences between individuals, we expect that many of them are either repetitive regions that were incorrectly collapsed during genome assembly or unresolved “gaps” in the genome assembly for which size was underestimated (Hehir-Kwa et al. 2016; Shi et al. 2016). Indeed, the fire ant genome includes >35% repetitive sequences (Li and Heinz 2000) (Suppl. Information 1.6), and such regions are difficult to resolve with the types of short-read technologies (Treangen and Salzberg 2011) used in the fire ant genome project.

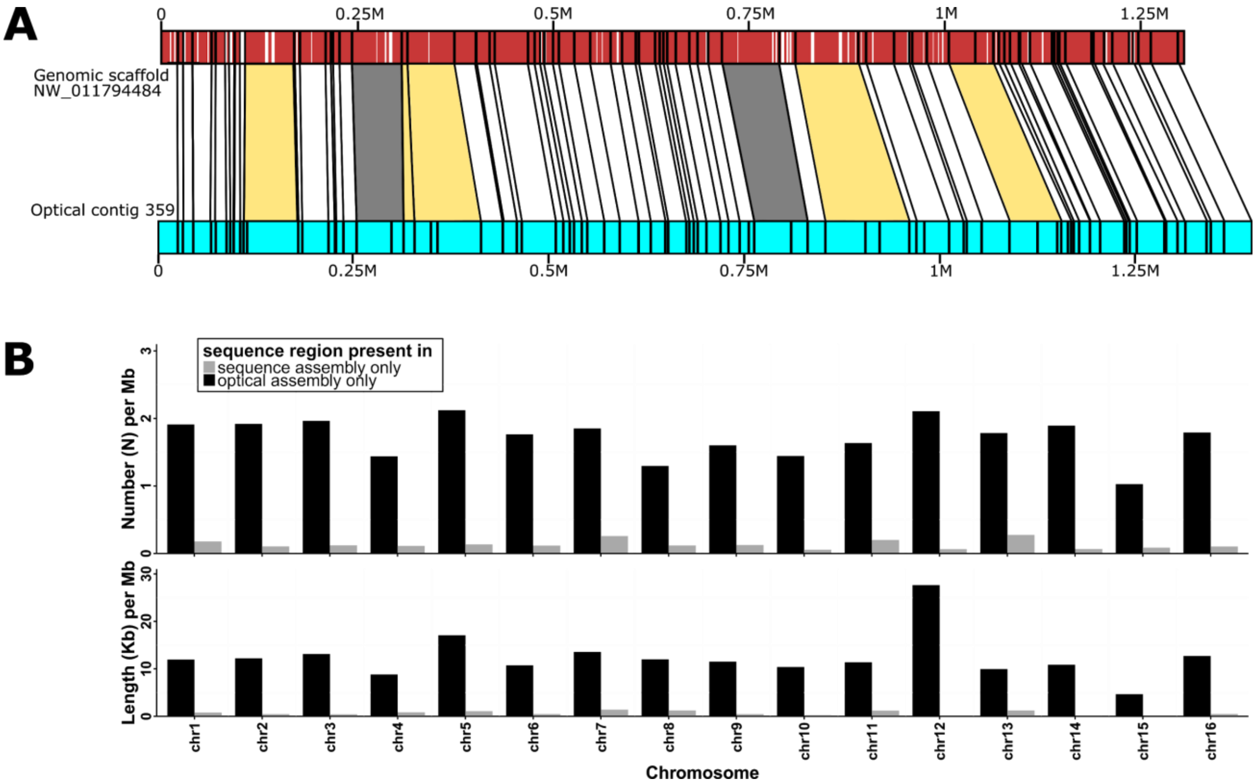

Suppl. Fig. S1. Comparison of the *S. invicta* reference sequence assembly to the *S. invicta SB* optical assembly. **A** Example of potential structural differences (grey: deletion, beige: insertion) between a scaffold of the *S. invicta* reference sequence assembly (top; brown) and a contig of the *S. invicta* optical assembly (bottom; blue), with stretches of undefined (N) nucleotides in white and *BspQ*I sites as black tick marks. **B** Plots showing for each chromosome the number and total length of regions present exclusively in either the *S. invicta* reference genome sequence (grey bars) or the optical assembly (black bars).

Suppl. Information 1.3. Large scale rearrangements detected by comparing optical assemblies

Rearrangements between SB and Sb in the three species

To find large-scale rearrangements between SB and Sb, we aligned each of the six the optical assemblies to the *S. invicta B* “core” reference optical assembly (produced in Suppl. Information 1.2). The alignments were done using BNG IrysView (v2.4) using alignment settings outlined in Suppl. Information 1.9. In the social chromosome (chromosome 16), none of the *B* samples showed evidence of any large scale rearrangement relative to this reference (Suppl. Fig. S2A). However, all *b* samples (*S. invicta, S. quinquecuspis, S. richteri*) had a contig that aligned to two *B* contigs mapping to separate locations approximately 10 Mb apart in the *B* chromosome-level assemblies (Suppl. Fig. S2A). This rearrangement could be interpreted as representing a translocation and subsequent inversion of a relatively small portion (approximately 305 kb) of the chromosome. However, the detected distal breakpoint co-locates with the end of the supergene region as identified from the pattern of SB-Sb sequence differentiation in the invasive north

American *S. invicta* population (Pracana et al. 2017). The more likely interpretation is thus that the rearrangement represents the single large inversion previously detected using BAC-FISH (Wang et al. 2013). Our result support the hypothesis that *S. quinquecuspis* and *S. richteri* also carry the same social chromosome supergene.

We found a second inversion in *S. invicta*, proximal to the large inversion, enclosed within an Sb optical contig that formed a one-to-one alignment with an SB optical contig (Suppl. Fig. S2). The raw comparison of optical assemblies indicated that this inversion spans 119 kb, with unaligned neighboring sequence. However, clearly matching optical markers flanking the inversion indicates that it in fact spans 1.74 Mb (Suppl. Fig. S2B). The discrepancy between the automatically detected inversion and what we conclude after manual curation of the alignment optical marker alignment can be attributed to limitations with the data, the degree of divergence between SB and Sb and algorithmic limitations which may struggle to perform optimal local realignment. In *S. richteri*, the optical assembly of this species was too fragmented to determine the presence or absence of this inversion. The 1.74 Mb inversion is located in a similar position to an even smaller (~48 kb) inversion reported previously (Wang et al. 2013). Unfortunately, the portion of the optical map spanned by this inversion has only three evenly spaced optical markers, making it impossible to detect whether this 48 kb inversion is present using optical reads.

The two rearrangements reported here support the hypothesis that rearrangements inhibit double-crossovers between SB and Sb that would otherwise occur in the middle of a single large inverted region (Stevison et al. 2011). These two rearrangements are located in the second half of the supergene region. The first half of the region was poorly assembled in all individuals (7.7 Mb to approximately 11.5 Mb of the optical chromosome 16). It is possible that this first half of the region includes additional recombination suppressing mechanisms, such as undetected rearrangements.

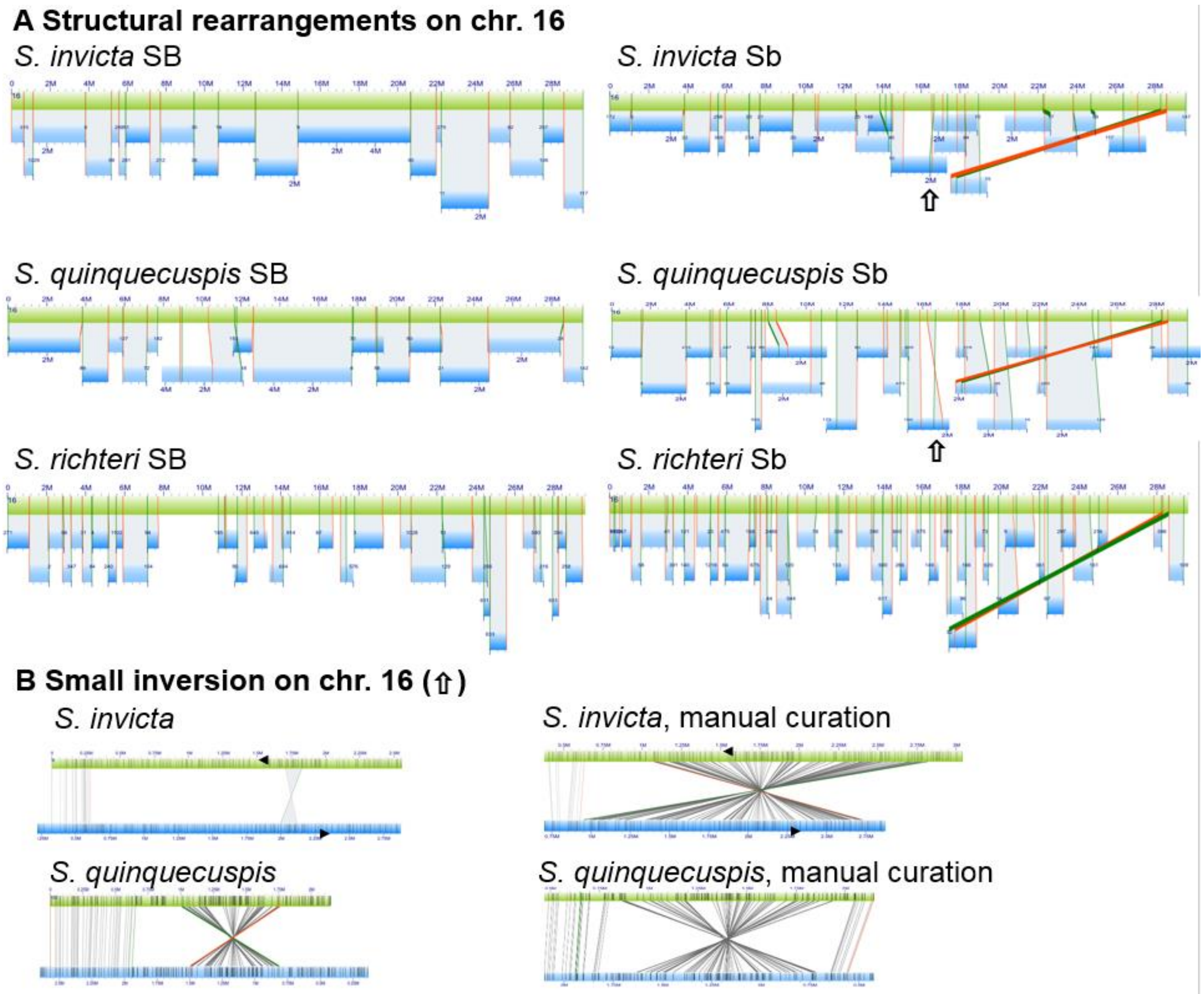

Suppl. Fig S2. Alignments of optical contigs and detected rearrangements between *Sb* and *SB*. Optical contigs (blue bars) were aligned to the optical chromosome 16 (*S. invicta* SB). **A** Overview of chromosome 16 which carries the social supergene. **B** Detailed view on the small inversion and its extent after manual curation.

To test whether the two rearrangements could represent different evolutionary "strata" representing steps in the expansion of the supergene (Lahn and Page 1999), we tested whether the amounts of neutral differentiation between SB and Sb differ between the two rearrangements. We used the previously published short read whole-genome sequences of 8 haploid *B* and 8 haploid *b* males collected from the North American *S. invicta* population (SAMN00014755, SRX206834 and SRP017317) (Wurm et al. 2011; Wang et al. 2013). We filtered the reads as previously reported (Pracana et al. 2017), then aligned them to the *S. invicta* reference assembly with bowtie2 (Langmead and Salzberg 2012) (v2.1.0) and called variants among the individuals using freebayes (Garrison and Marth 2012) (v1.0.2-33-gdbb6160). We discarded any variant with QUAL<=20. To filter the gene set, we removed any gene that had the tags "partial=true" or "the sequence of the model RefSeq transcript was modified relative to this genomic sequence to represent the inferred CDS"; we similarly removed genes with very high or very low coverage in any of the two groups of individuals (indicative of putative misassemblies, deletions or duplications), for which the coding sequence either

included an N character in the reference genome assembly or for which the length was not a multiple of three in any of the individuals. The resulting gene set includes 394 high quality genes out of the 443 in the supergene region. For each gene in the supergene region, we created a consensus for SB and another for Sb based on the fixed substitutions between the two groups of individuals, using bcftools consensus (Li 2011) (v1.3.1), then measured the rate of synonymous substitutions (dS) between the two groups using the R package seqinR (Charif and Lobry 2007).

The amount of neutral differentiation did not differ between the two rearrangements (mean dS = 3.0×10^{-3} in the large inversion, dS = 2.5×10^{-3} in the small inversion; t-test, $t_{d.f.=36.2} = 0.74$, p = 0.47; Suppl. Fig. S3), thus it is unlikely that each represents a step in the expansion of the supergene. This absence of evolutionary strata is consistent with a previous report that there are no strata of differentiation between SB and Sb in the assembled portion of the supergene in terms of single nucleotide polymorphisms in North American *S. invicta* (Pracana et al. 2017). The first half of the chromosome is mostly absent from the reference genome assembly, thus it was impossible to test whether the two halves of the supergene represent different evolutionary strata.

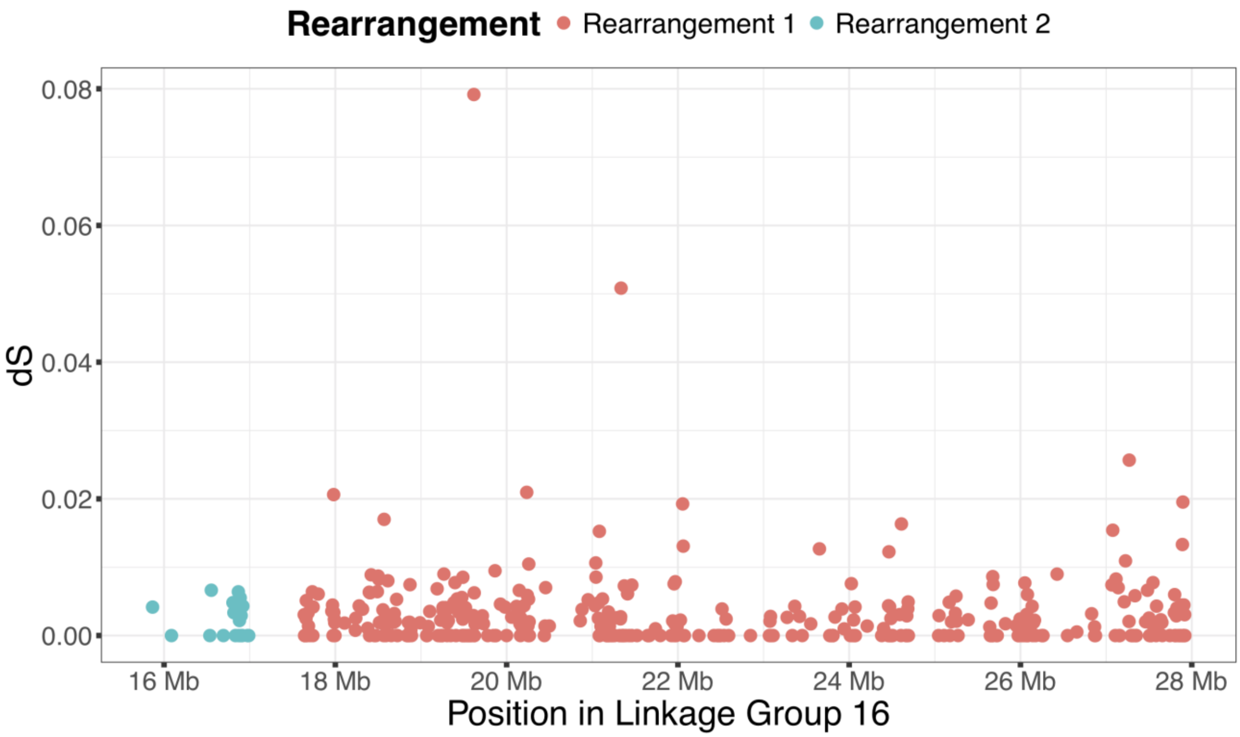

Suppl. Fig. S3. The rate of synonymous substitution (dS) between SB and Sb in *S. invicta* reference assembly scaffolds in the genes mapped to the 1.74 Mb inversion ("Rearrangement 1") and the 10.5 Mb inversion ("Rearrangement 2").

Additional rearrangements and inversions in the genomes of the three fire ant species

We performed pairwise comparisons between the optical assemblies of all samples to determine whether there are any additional rearrangements. We found that the *S. quinquecuspis B* sample

carries a 181 kb region that is inverted in comparison to the *S. quinquecuspis b* sample (Suppl. Fig. S4). It would be necessary to sequence multiple individuals of each genotype per species to determine whether this third rearrangement is a segregating polymorphism in this species or in multiple species, or whether it is fixed in SB among *S. quinquecuspis* individuals.

To determine whether this region is also inverted in other samples, we performed additional comparisons (not shown):

1. *S. invicta* Sb (compared to *S. invicta* SB): region aligns; no inversion.
2. *S. richteri* SB (compared to *S. invicta* SB): region aligns; no inversion.
3. *S. richteri* Sb (compared to *S. quinquecuspis* Sb): region aligns; no inversion.
4. *S. quinquecuspis* SB (compared to *S. invicta* SB): the region of does not align. This is consistent with the presence of an *S. quinquecuspis* SB-specific inversion.

We thus conclude that within our dataset, this inversion is specific to *S. quinquecuspis* SB.
We found evidence for large-scale rearrangements outside the supergene region (Suppl. Fig. S4). We detected two translocations on chromosome 3, one in both the *S. quinquecuspis B* and *b* samples relative to the samples of the other two species (465 kb) and another in the *S. richteri B* sample (470 kb, which may represent an inversion, based on the orientation of the alignment of the optical contig supporting the translocation; *S. richteri b* was too fragmented to confirm the presence of this rearrangement). We also detected two inversions. The first spanned 700 kb on chromosome 7 seen in the *S. quinquecuspis B* individual (*S. quinquecuspis b* was too fragmented in the region spanned by the rearrangement to confirm its presence in this sample). The second inversion spanned 400 kb on an unplaced optical contig of the *S. richteri B* individual (*S. richteri b* was too fragmented to confirm the presence of this rearrangement).

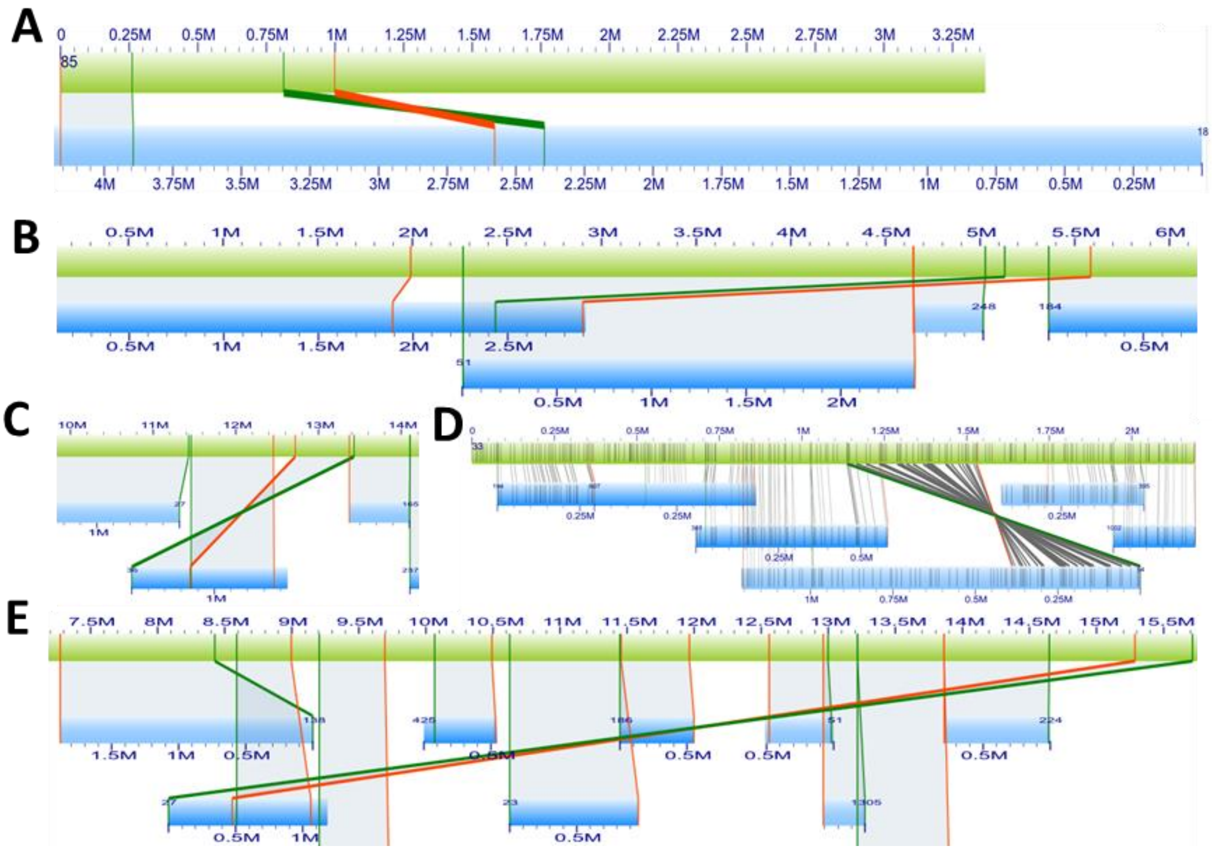

Suppl. Fig. S4. Further rearrangements detected in fire ants. **A** Small inversion in *S. quinquecuspis* Sb chr. 16 (181308 bp, optical contig 85, 0.8-1 Mb) (compared to *S. quinquecuspis* SB, optical contig 18, 2.5 Mb). This small inversion was not detected in any other sample. **B** Translocation in chromosome 3 of the *B* and *b* samples of *S. quinquecuspis* relative to the *S. invicta B* sample (465 kb); **C** Inversion on chromosome 7 of the *S. quinquecuspis B* sample relative to *S. invicta B* (700kb); **D** Inversion between the *S. richteri B* and the *S. quinquecuspis B* samples on an unplaced optical contig; **E** Translocation and putative large inversion in chromosome 3 of *S. richteri B* relative to *S. invicta B.*

Suppl. Information 1.4. Detection of insertions and deletions from optical mapping data

Insertions and deletions flanked on both sides by aligning sequence

We conducted pairwise alignments between all samples with BNG IrysView (v2.4) using alignment settings outlined in Suppl. Information 1.9. We then used a previously published script (https://github.com/RyanONeil/structome) (Kawakatsu et al. 2016) to detect large indels between each pair of individuals. This method only detect indels located inside the alignment between two contigs, and not any alignment that affects either end of one of the contigs. To minimize false-positive detection, we used a 3 kb minimal size requirement for indels (Kawakatsu et al. 2016). The majority (72%) of the detected indel differences were in optical contigs which we could place on chromosomes. Further summaries and visualization of the detected indels were computed in R (R Core Team 2013). For the pairwise comparison of *S. invicta* optical assemblies (*B* and *b*), we performed a comparison of indels detected in reciprocal alignments (*b vs B* compared with *B vs b*). Both comparisons yielded nearly identical results (95% of sites were recovered; data not shown), indicating high consistency of indel detection.

We detected 226 insertions with a total length of 3.05 Mb and 306 deletions with a total length of 3.09 Mb between S. invicta *b* and *B* individuals (Suppl. Table S2, Suppl. Table S3). Indel sizes ranged from 3 kb to several hundred thousand kb (Suppl. Fig. S5). Indels detected between *b* and *B* individuals of *S. quinquecuspis* and *S. richteri* are shown in Suppl. Table S3 along with indels found in interspecific comparisons.

Suppl. Table S2. Insertions and deletions (indels) in the *b* sample relative to the *B* sample of the fire ant *S. invicta*.

**Chromosome length (Mb)**	**Social Chr.**	**Other Chr.**	**Unplaced**
B sample	29.61	321.33	65.50
b sample	36.44	310.76	70.00
**Insertions/Deletions [ratio]**			
Number	55/14	108/173	63/119
	[3.93]	[0.62]	[0.53]
Density (number/Mb)	1.86/0.47	0.34/0.54	0.97/1.84
	[3.96]	[0.63]	[0.53]
Total length (Mb)	1.43/0.11	1.01/1.63	0.61/1.35
	[13.00]	[0.62]	[0.45]
Chromosome length	[1.23]	[0.97]	[1.10]

Suppl. Table S3. Summary of detected Insertions and Deletions (>3kb) in pairwise comparisons between the samples.

Query	*invicta b*	*quinquecuspis b*	*richteri b*	*quinquecuspis B*	*richteri B*	*richteri B*	*quinquecuspis b*	*richteri b*	*richteri b*
Ref	*invicta B*	*quinquecuspis B*	*richteri B*	*invicta B*	*quinquecuspis B*	*invicta B*	*invicta b*	*quinquecuspis b*	*invicta b*
Comparision type (specific)	intra	intra	intra	inter (*B*)	inter (*B*)	inter (*B*)	inter (*b*)	inter (*b*)	inter (*b*)
Total Length of Deletions	3090846	1896776	3910234	6681980	3192190	3446416	4732612	4759061	4510515
Total Length of Insertions	3051408	2697340	1754108	7236631	6358073	6173108	6985565	3526040	3992533
Total numbers of Deletions (in query)	306	159	771	543	324	349	407	550	420
Total numbers of Insertions (in query)	226	252	109	506	743	641	701	316	401
Chr. 16 insertions	55	53	50	23	55	45	48	27	44
Chr. 16 deletions	14	13	41	37	20	22	45	63	57

For *S. invicta*, the distribution of insertions was not homogeneous across the 16 chromosomes (χ^2 test for given probabilities, $\chi^2 = 151.58$, d.f. = 15, $p < 10^{-23}$). This bias was driven by the social chromosome (Suppl. Fig. S6), the only chromosome to have a significant enrichment of insertions (Z-score = 11.12, Bonferroni-corrected $p < 10^{-26}$; for all other chromosomes $p > 0.05$). The distribution of deletions was homogeneous across the 16 chromosomes (χ^2 test for given probabilities, $\chi^2 = 24.02$, d.f. = 15, $p = 0.065$).

For *S. quinquecuspis*, the distribution of insertions was similarly not homogeneous across the 16 chromosomes (χ^2 test for given probabilities, $\chi^2 = 104.89$, d.f. = 15, $p < 10^{-14}$). Again, this bias was driven by the social chromosome, the only chromosome to have a significant enrichment of insertions (Z-score = 8.71, Bonferroni-corrected $p < 10^{-16}$; for all other chromosomes $p > 0.05$). In this species, the distribution of deletions was not homogeneous across the 16 chromosomes (χ^2 test for given probabilities, $\chi^2 = 25.47$, d.f. = 15, $p = 0.04$). This heterogeneity was weak: no chromosome had a statistically significant enrichment of deletions (Bonferroni-corrected $p > 0.05$ for all chromosomes), but chromosomes 10 and 14 had low residual values (-2.32 and -2.07, respectively).

For *S. richteri*, the distribution of insertions was similarly not homogeneous across the 16 chromosomes (χ^2 test for given probabilities, $\chi^2 = 230.89$, d.f. = 15, $p < 10^{-39}$). Again, this bias was driven by the social chromosome, the only chromosome to have a significant enrichment of insertions (Z-score = 14.07, Bonferroni-corrected $p < 10^{-43}$; for all other chromosomes, $p > 0.05$). In this species, the distribution of deletions was not homogeneous across the 16 chromosomes (χ^2-squared test for given probabilities, $\chi^2 = 33.35$, d.f. = 15, $p = 0.004$). This bias is driven by chromosome 1 (Z-score = 3.88, Bonferroni-corrected $p = 0.002$; for all other chromosomes $p > 0.05$). *S. richteri* showed a high number of small deletions (Suppl. Table S3, Suppl. Fig. S5) which likely are a result of the short average contig length, thus high fragmentation of the optical assemblies of both the *b* and the *B* individual (Suppl. Table S1). This is a technical limitation causing difficulties for the alignment and a higher likelihood to detect deletions than insertions.

Taking the difference in length between the insertions and deletions showed that in *S. invicta*, the *b* individual had 44.51 kb more sequence per Mb in the social chromosome than the *B* individual, compared to a mean of 2.17 kb less sequence per Mb (± standard deviation of 4.34 kb per Mb) in the rest of the genome (Suppl. Fig. S6). In *S. quinquecuspis*, the *b* individual had 22.37 kb more sequence per Mb in the social chromosome than the *B* individual, compared to a mean of 0.57 kb less sequence per Mb (± s.d. of 7.07 kb per Mb) in the rest of the genome. In *S. richteri*, the *b* individual had 7.32 kb more sequence per Mb in the social chromosome than the *B* individual, compared to a mean of 7.32 kb less sequence per Mb (± s.d. of 1.97 kb per Mb) in the rest of the genome.

Outside the social chromosome, we observed higher numbers of indels between species than within species (Suppl. Table S2, Suppl. Table S3, Suppl. Fig. S6). Interspecific comparisons of indel frequencies (*B* vs *B* and *b* vs *b*) showed that the chromosome 3 is enriched for large sequence regions present in *S. richteri* in comparison to *S. invicta* or *S. quinquecuspis* (highly significant deviation from expected distribution in comparisons to any other *Solenopsis* species as per χ^2 test, with highest residuals for chr. 3). We observed a similar pattern for chromosome 1 of *S. quinquecuspis*, but this was only only significant in the *S. quinquecuspis b* male compared to *S. invicta*.

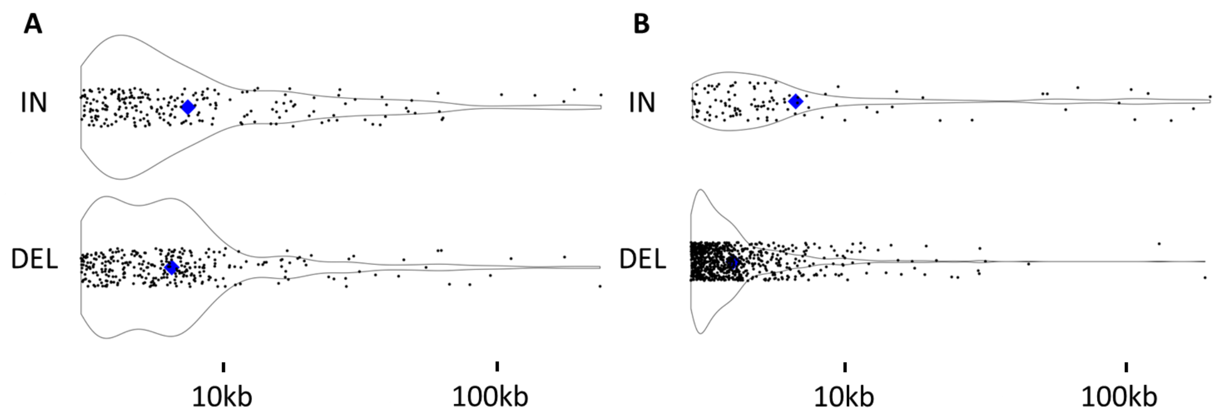

Suppl. Fig. S5. Length distribution of indels. **A** Indels detected between *S. invicta b* and *B* individuals. The blue diamond indicates the mean length for insertions ("IN", top) in *b* compared to *B* and deletions ("DEL", bottom) from *b* compared to *B*. **B** Indels detected between *S. richteri b* and *B* individuals. The blue diamond indicate the mean length for insertions ("IN", top) in *b* compared to *B* and deletions ("DEL", bottom) in *b* compared to *B*. Note the bias towards small deletions due to low optical assembly quality.

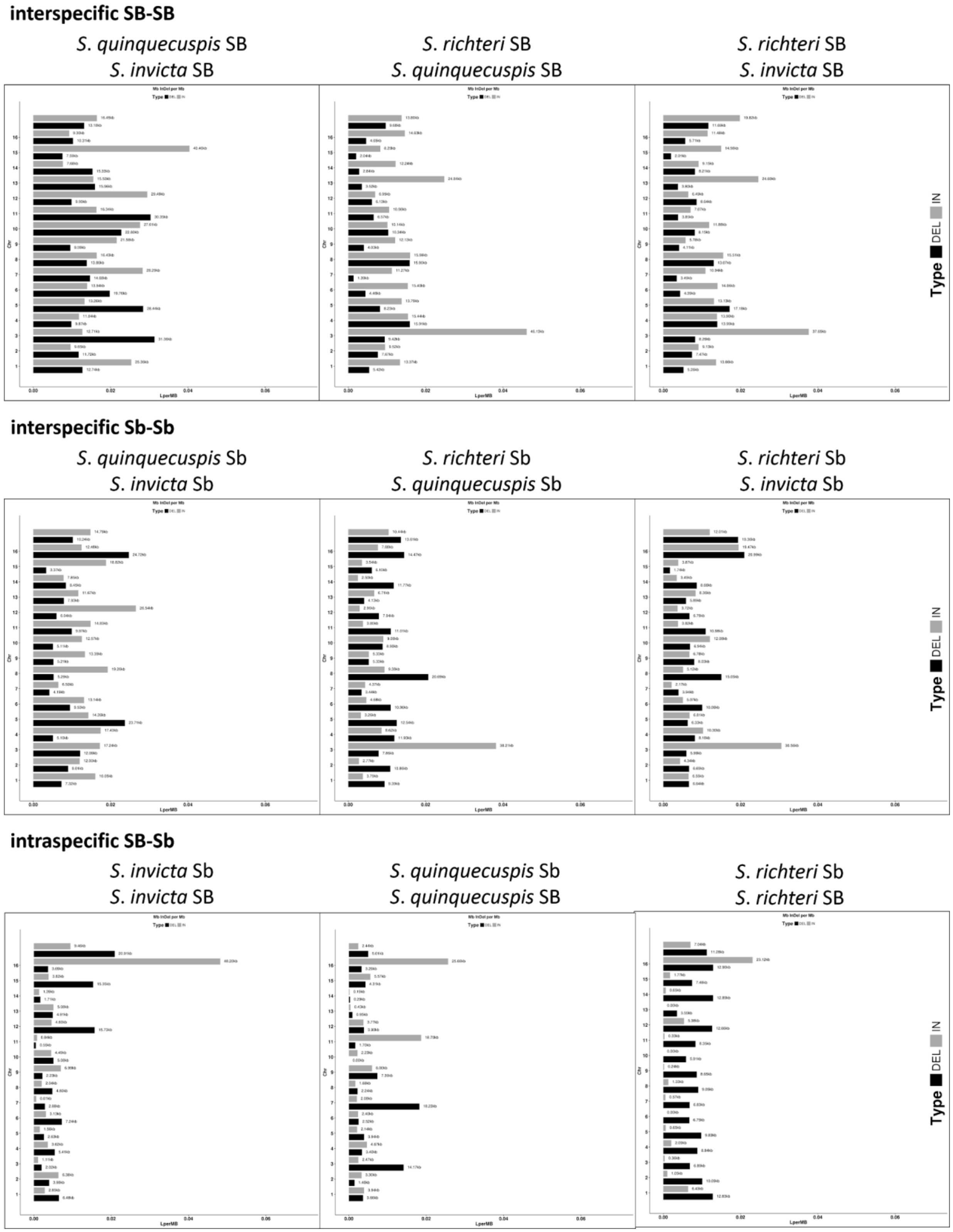

Suppl. Fig. S6. Visualization of indel distribution per chromosome for intra-and inter-specific comparisons, based on direct pairwise comparisons of optical maps to identify large (>3kb) insertions (grey) and deletions (black) within and between species and variants of the social chromosome. The y-axes display from bottom to top: chromosomes 1 to 16, and, in addition, unplaced contigs.

"Overhanging" insertions and deletions between *B* and *b*

The method reported above can only detect indels which are within a single contig in each assembly and flanked by aligning regions. Any indel that affects either end of one of the contigs will not appear as an indel. Instead, it will be seen as sequence present at the 3' or 5' end of a contig in one of the samples, but not on the matching contig in the other sample (Suppl. Fig. S7). We performed additional analyses to detect these "overhanging" indels in a pairwise comparison between the *B* and *b* samples of *S. invicta*, which have the most contiguous assemblies. We did not perform this analysis for the other two species, because their lower assembly contiguity (Suppl. Table S1) would confound results.

We detected overhangs in *b* and *B* individuals in almost every chromosome (Suppl. Table S4). The distribution of overhangs in the *b* assembly compared to the *B* assembly is not homogeneous across the 16 chromosomes (χ^2 test for given probabilities, $\chi^2 = 36.66$, df = 16, p = 0.002). This is driven by the social chromosome (Z-score = 3.48, Bonferroni-corrected p = 0.009; for all other chromosomes p > 0.05, Suppl. Fig. S7). Indeed, the supergene region of the social chromosome includes only 5.96% of the assembly (20.91 Mb out of 350.94 Mb in 16 optical chromosomes), but includes 15.12% of the *b* overhangs (13 out of 86), cumulatively representing 77.41% of the inserted sequence (11.25 Mb out of 14.53 Mb, χ^2, supergene: Bonferroni-corrected $p < 10^{-18}$, all other chromosomes p > 0.05, Suppl. Fig. S7).

The distribution of overhangs in the *B* assembly compared to the *b* assembly is homogeneous across the 16 chromosomes and the supergene (χ^2 test for given probabilities, $\chi^2 = 20.37$, df = 16, p = 0.2). The length of overhangs was biased towards the supergene (supergene: Bonferroni-corrected $p < 10^{-27}$, all other chromosomes p > 0.05). The supergene carried 5.98 Mb overhangs (29.78% of 20.07 Mb overhangs in total, Suppl. Fig. S7).

Thus, in the social chromosome the *b* individual carried 5.27 Mb (17.80%) more sequence than the *B* individual. To put this in other words, *b* had 252.03 kb more sequence per Mb in the social chromosome supergene than the *B* individual, compared to a mean of 33.86 kb less sequence per Mb in the rest of the genome (standard deviation = 19.70 kb per Mb) (on average 0.72 Mb (13.62%, from 1.43 Mb to 0.25 Mb, Suppl. Table S4) size reduction per chromosome in the *b* individual). The difference in size change in the b individual chromosome 16 is highly significant (non-homogeneous distribution of size changes, χ^2 test for given probabilities, $\chi^2 = 83.25$, df = 15, $p < 10^{-10}$; driven by chromosome 16, Z-score = 8.12, Bonferroni-corrected $p < 10^{-14}$, for all other chromosomes p > 0.05).

Suppl. Table S4. Overhangs detected (numbers and length) per chromosome in *b* and *B* individuals of *S. invicta*.

Chromosome	Number of overhangs in *B* compared to *b*	Number of overhangs in *b* compared to *B*	Cumulative lengths (Mb) of overhangs in *B* compared to *b*	Cumulative lengths (Mb) of overhangs in *b* compared to *B*	Length in *b* compared to *B*	
					(Mb)	(%)
1	6	6	0.969	0.212	-0.756	-2.23
2	8	6	0.616	0.240	-0.376	-1.33
3	8	8	1.723	0.597	-1.127	-4.53
4	4	1	0.595	0.029	-0.566	-2.10
5	4	2	0.264	0.011	-0.253	-1.11
6	5	3	0.813	0.093	-0.720	-2.76
7	7	6	0.966	0.185	-0.781	-3.90
8	5	6	1.039	0.099	-0.940	-3.69
9	3	0	0.444	0	-0.444	-2.71
10	5	4	0.868	0.508	-0.360	-2.00
11	9	9	1.699	0.270	-1.429	-7.08
12	4	6	1.120	0.283	-0.837	-5.21
13	8	7	1.486	0.600	-0.885	-6.10
14	6	3	0.918	0.034	-0.884	-5.64
15	7	5	0.573	0.121	-0.452	-3.85
16	8	14	5.978	11.247	5.269	17.80

The reduction in size in *b* chromosomes 1 to 15 is due to the slightly higher fragmentation and on average shorter optical contig length of the assembly of the *b* individual (see Suppl. Table S1). This is evidenced by a lower density of optical markers in the *B* overhangs (average of 2.80 optical markers / 100 kb for chr 1-15, while the average optical marker density / 100 kb is 8.31) (Suppl. Fig. S7). The optical marker density determines the alignability between optical contigs and also strongly influences the assembly contiguity. Low density regions require high numbers of very long optical molecules spanning the region in order to allow assembly. Thus, less and shorter Sb optical reads (compared to SB) inevitably lead to fewer low-marker-density regions being present in Sb optical contigs. However, the *B* overhangs within the supergene region was 7.13 optical markers / 100 kb (Suppl. Fig. S7), close to the average. This suggests that the majority of *B* overhangs in chromosomes 1 to 15 are due to technical limitations of the *b* optical assembly, while in the supergene region they may stem from true differences, *i.e.* due to large indels, sequence divergence and/or chromosomal rearrangements.

Total change in chromosome sizes in the b individual

Considering that chromosome size changes are the sum of the overhangs and the indels, the total change in chromosome sizes in the *b* individual compared to the *B* individual is on average -3.83% (-0.762 Mb) for chromosomes 1 to 15, and 22.25% (6.588 Mb) for the social chromosome, whereby the supergene on its own changed by 31.68% (6.624 Mb) (Suppl. Fig. S7).

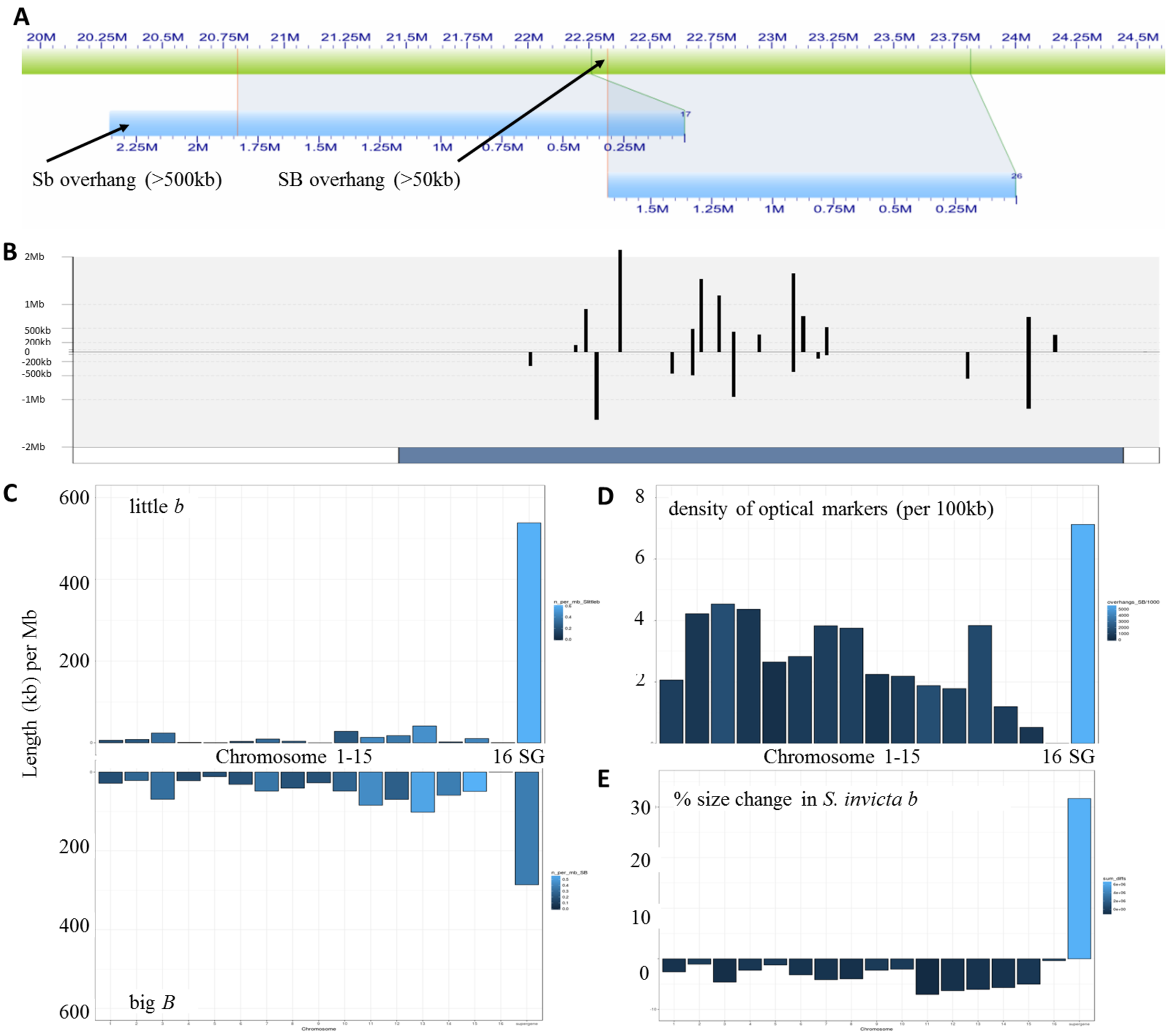

Suppl. Fig. S7. Analysis of overhangs in *S. invicta b* and *B* individuals. **A** Example of overhangs (highlighted using arrows) in comparisons between *S. invicta B* and *b*, representing putative indels which do not fall within an alignment. **B** Distribution of overhangs in *S. invicta* Sb (positive y-axis values) and SB (negative y-axis values) across chromosome 16. The blue bar indicates the supergene region. **C** Density of *b* overhangs (positive) and *B* overhangs (negative) (length in kb per Mb for chromosome 1 - 16 and the supergene (SG)). **D** Density of optical markers (optical markers per 100 kb) in *B* overhangs, coloured by *B* overhang length (in kb) for chromosome 1 - 16 and the supergene. **E** Total size changes by indels and overhangs (%) in chromosomes 1-15, chromosome 16 (regularly recombining part), and the supergene of chromosome 16 (here shown as 17th chromosome "SG") in the *S. invicta b* individual, compared to the *S. invicta B* individual. The colouration is by total (bp) change of size.

Suppl. Information 1.5. K-mer based estimation of genome sizes of 10 haploid males

We sequenced the genomes of 5 pairs of one *B* and one *b* male of *S. invicta* from Brazil (two pairs from neighboring colonies in Campo Grande (-20.48, -54.75), two pairs from the same colonies in Miranda (-20.13, -56.69), 1 pair from the same colony in General Camara (-29.92, -51.76)) on an Illumina HiSeq4000 (2*150 bp, TruSeq PCRfree library preparation, 350 bp insert size; raw reads are available under NCBI BioProject PRJNA396161). We first performed quality filtering and adapter trimming using skewer (Jiang et al. 2014) (v0.2.2, minimum window base quality of 20, minimum end-quality of 15, minimum length of 100, filter against degenerated N containing reads). To deplete the remaining reads of DNA that is not nuclear *S. invicta*, we aligned (bwa mem (Li and Durbin 2010), v0.7.17-r1188, parameters: -B 20 -O 20 -E 20 -L 100 -U 100) each sample to mt-DNA (NC_014672.1 *Solenopsis invicta*), *Wolbachia* DNA (fragments from *S. invicta Wolbachia* type A and B: AF243435.1 Wolbachia_wSinvictaA_wsp, AF243436.1 Wolbachia_wSinvictaB_wsp; full genomes of *Wolbachia* type A and B: CP001391.1 Wolbachia_sp_wRi, AM999887.1 Wolbachia_Culex_quinquefasciatus_Pel_strain_wPip) and phiX phage (NC_001422.1, Illumina spike-in control). Alignments were processed and filtered in parallel using sambamba (Tarasov et al. 2015) (v0.6.7, filter parameter: -F "paired and (not (mapping_quality >= 45 and sequence_length >= 80) or supplementary) or unmapped)") and samtools (Li et al. 2009) (v1.6, fixmate). We then extracted unmapped reads and those with supplemental alignments or mapping score smaller than 45 using bedtools (Quinlan and Hall 2010) (v2.27.1, bamtofastq). We discarded from these reads any smaller than 140 bp using seqtk (v1.2-r102-dirty, https://github.com/lh3/seqtk), then trimmed remaining reads to 140 bp (fastx toolkit, v0.0.13, http://hannonlab.cshl.edu/fastx_toolkit) and merged forward and reverse reads. We normalized (subsampled with seqtk, v1.2-r102-dirty) each sample to 48,463,635 reads. For each sample, we measured the k-mer frequency distribution using KMC3 (Kokot et al. 2017) (v3.0.0, k=15 to k=41 in k=2 steps, canonical k-mers, no upper frequency limit), from which we measured genome size and repetitive genomic fractions using findGSE (Sun et al. 2018). The highest k at which the analysis converged in all samples and the repeat content estimate mirrored an independent measure (Suppl. Information 1.6) was k=21, thus this value was selected for the further comparison (individual k-mer frequency distribution plots can be found in Suppl. Information 9). Results from higher k for converging individual samples were consistent with the findings from k=21. A paired t-test was then used to compare the genome size and repeat content estimates of the *B* and *b* individuals in R.

The genome sizes estimated for *b* samples were 3.59% larger (95% confidence interval: 2.02% to 5.16%) than those of *B* samples (paired one-sided t-test: $p < 0.002$; Fig. 1C, Suppl. Table S5). The genomes of the *b* samples included 4.55% more repetitive sequence (0.52% to 8.57%) than the *B* samples (paired one-sided t-test: $p < 0.018$, Suppl. Table S5).

Suppl. Table S5. K-mer-based estimates for genome size and repeat content of *S. invicta b* and *B* male individuals (see also Fig. 1c).

Sample_pair	Size *B*	Size *b*	Repeat *B*	Repeat *b*
1. Mir6B_vs_Mir6b	447.815	468.134	0.35	0.36
2. GCa8B_vs_GCa8b	459.866	476.849	0.36	0.38
3. Mir1B_vs_Mir1b	434.878	448.927	0.33	0.36
4. CGI1B_vs_CGIn5b	453.628	461.154	0.36	0.37
5. CGIn3B_vs_CGIn11b	454.796	475.943	0.36	0.38

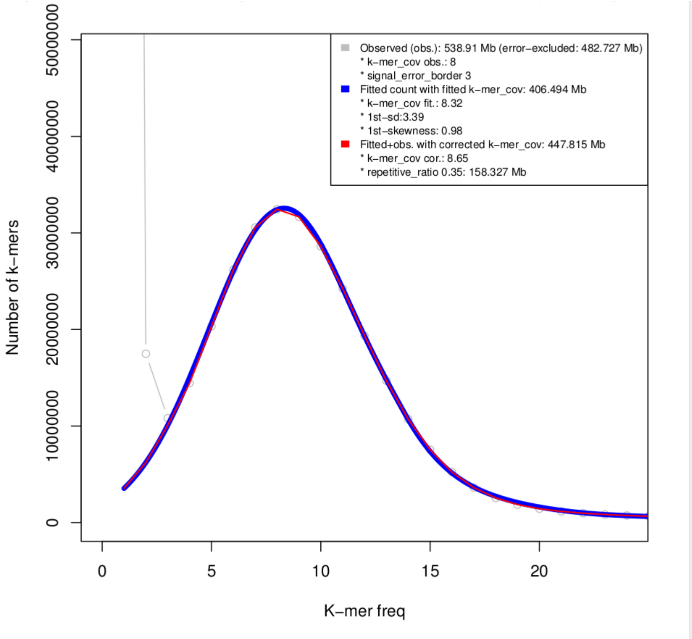

Suppl. Fig. S8.1. K-mer frequency histogram and model fit (findGSE) to determine the genome size of individual Mir6 *B*.

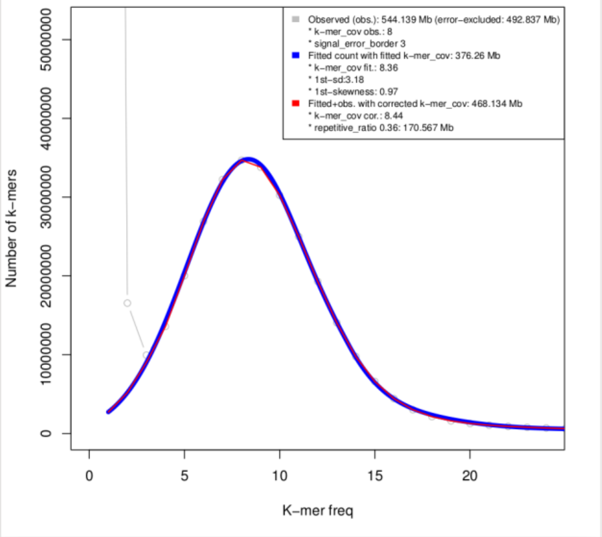

Suppl. Fig. S8.2. K-mer frequency histogram and model fit (findGSE) to determine the genome size of individual Mir6 *b*.

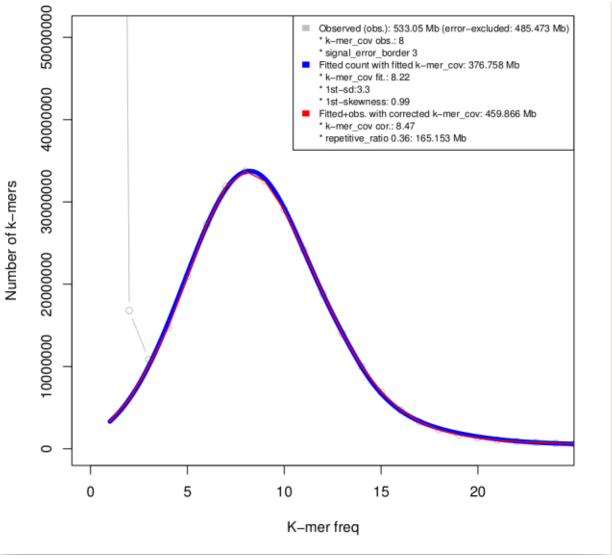

Suppl. Fig. S8.3. K-mer frequency histogram and model fit (findGSE) to determine the genome size of individual GCa8 *B*.

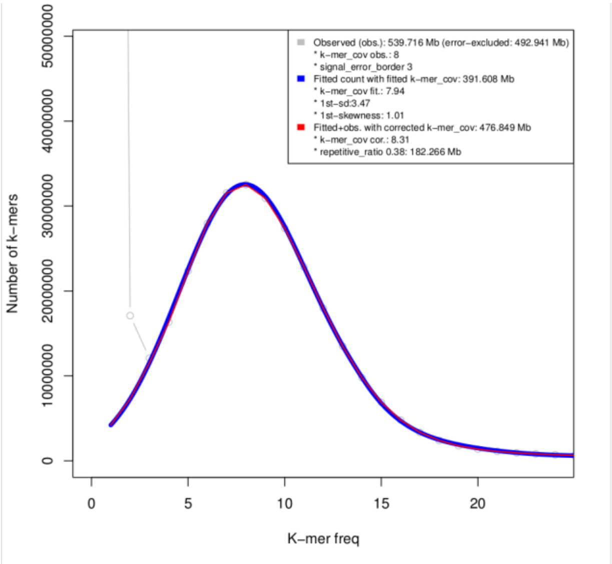

Suppl. Fig. S8.4. K-mer frequency histogram and model fit (findGSE) to determine the genome size of individual GCa8 *b*.

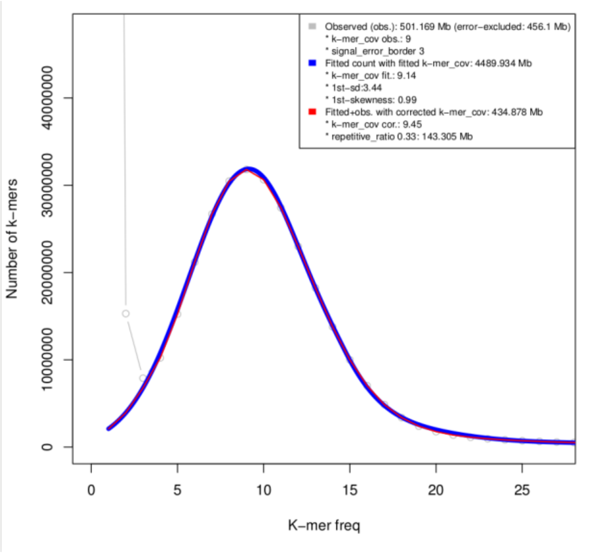

Suppl. Fig. S8.5. K-mer frequency histogram and model fit (findGSE) to determine the genome size of individual Mir1 *B*.

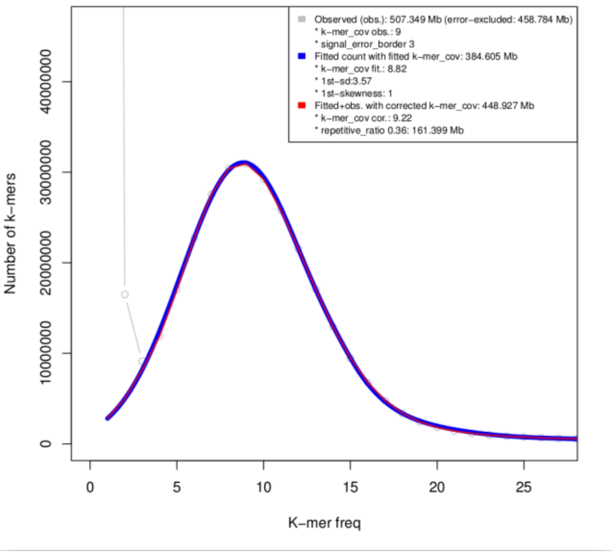

Suppl. Fig. S8.6. K-mer frequency histogram and model fit (findGSE) to determine the genome size of individual Mir1 *b*.

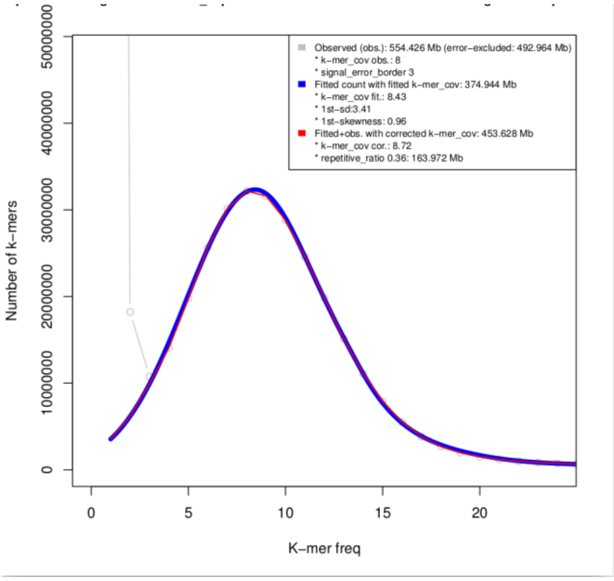

Suppl. Fig. S8.7. K-mer frequency histogram and model fit (findGSE) to determine the genome size of individual CGI1 *B*.

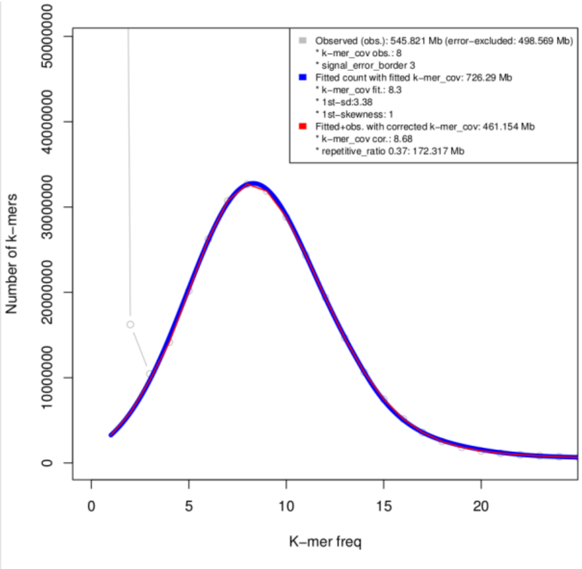

Suppl. Fig. S8.8. K-mer frequency histogram and model fit (findGSE) to determine the genome size of individual CGIn5 *b*.

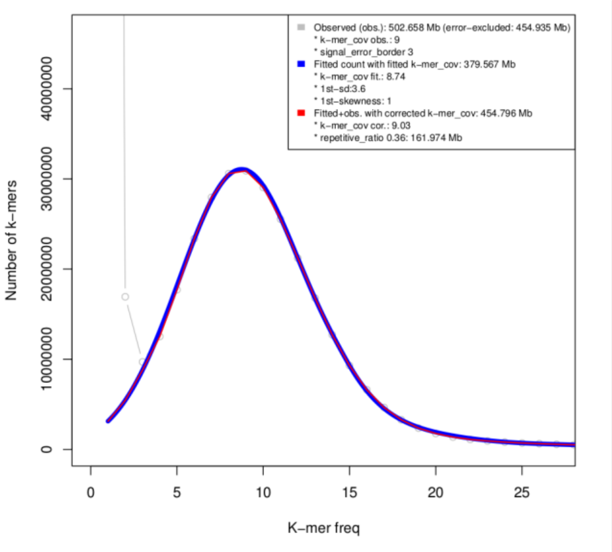

Suppl. Fig. S8.9. K-mer frequency histogram and model fit (findGSE) to determine the genome size of individual CGIn3 *B*.

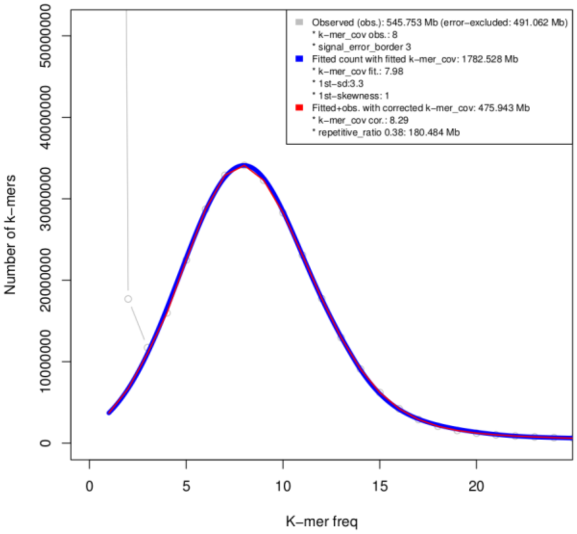

Suppl. Fig. S8.10. K-mer frequency histogram and model fit (findGSE) to determine the genome size of individual CGIn11 *b*.

Suppl. Information 1.6. Comparison of repeat content between individuals carrying alternate variants of the supergene

Repeats detected from short paired-end reads

Using the identical normalized and length-trimmed reads from the same paired individuals as used for the k-mer-based analysis, we performed a reference-independent repeat analysis. We analyzed genomic repeat content and repeat types with DNApipeTE (Goubert et al. 2015) (v1.2, based on a genome size of 450 Mb) and repbase (Bao et al. 2015) (v22.12) with integration of *S. invicta* centromeric satellite repeats (Huang et al. 2016). We ran DNApipeTE with 4 iterations during which reads were subsampled to represent 0.3X average genomic short read coverage, then assembled and annotated.

The estimated total repeat content was 2.23% higher (95% confidence interval 1.41% - 3.04%) in *b* than *B* individuals within pairs (paired one-sided t-test: $p < 0.0009$) (Suppl. Table S6). A full list of repetitive elements for each individual can be found in supplementary table S8 (separate supplementary file in the supplementary material online).

Suppl. Table S6. DnaPipeTE-based estimates of total genomic repeat content.

Sample_pair	Repeats *B*	Repeats *b*
1. Mir6B_vs_Mir6b	0.3575	0.3739
2. GCa8B_vs_GCa8b	0.3747	0.3893
3. Mir1B_vs_Mir1b	0.3473	0.3719
4. CGI1B_vs_CGIn5b	0.3613	0.3915
5. CGIn3B_vs_CGIn11b	0.3543	0.3798

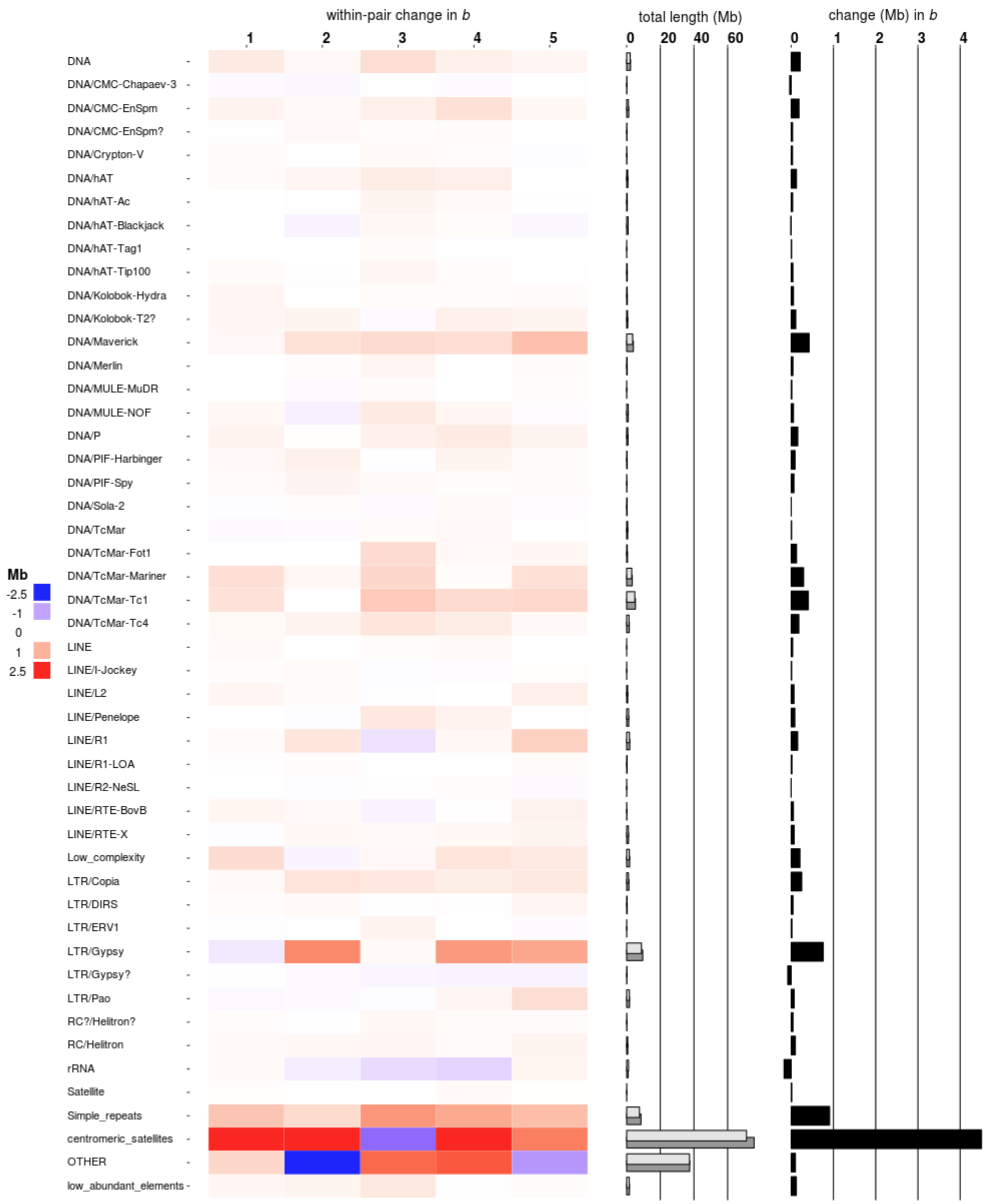

Suppl. Fig. S9. Analysis of repetitive elements in five pairs of *B*-*b*. The heatmap hows the change (Mb) in the *b* individual within each pair for those elements showing at least a change of 50 kb in at least 1 pair. The bar plots show average (over 5 pairs) values for total length per element in *B* (light grey), *b* (dark grey) and average change in *b* (black) in Mb.

The absolute increase in DNA content by all detected repeats in *b* individuals was on average 10.02 Mb (6.57 Mb - 13.58 Mb). However the pattern differed between classes of repeats. Simple repeats had consistently higher prevalence in *b* (increase of 0.47 Mb to 1.12 Mb in each *b* compared to the paired *B*, Suppl. Fig. S9)). The same was true for nonautonomous DNA elements, and transposable elements DNA/CMC-EnSpm, DNA/Maverick, DNA/TcMar-Fot1, DNA/TcMar-Mariner and LTR/Copia (Suppl. Fig. S9), each of which further had a prevalence of at least 300 kb more in *b* than *B* in at least one of the pairs. The following repetitive elements consistently had higher prevalence in *b*, but to a much lower extent (<300 kb total length increase in *b*): DNA/hAT, DNA/Kolobok-Hydra, DNA/P, DNA/PIF-Spy, DNA/PiggyBac, DNA/TcMar-Tc4, LTR/ERV4 and RC/Helitron.

Other repetitive elements showed substantial (>300 kb) *b* increases in specific, pairs, but slight decreases in other pairs: LINE/R1, DNA/TcMar-Tc1, Low_complexity, LTR/Gypsy, LTR/Pao, CenSol Satellites and unspecified “other” elements (Suppl. Fig. S9). In particular, the centromeric satellite repeats (CenSol) were massively more frequent in the *b* individuals in two of the pairs (9.38 Mb and 10.48 Mb increase), but showed a weaker increase (2.70 Mb and 1.65 Mb) or slight decrease (-1.68 Mb) in the other three pairs (Suppl. Fig. S9).

Our results show that *b* individuals are characterized by an overall increase of repetitive content, but that there is extensive variation between pairs of individuals with regards to the specific repetitive elements driving the overall increase. Individual variability in the amount of specific repeats was also reported for the platypus Y chromosome (Kortschak et al. 2009). Such individual variance could originate from each *B*/*b* pair having substantial specific history during which there was variable activity of transposases or duplications of normal genomic fragments such as reported in liverwort (Ishizaki 2002). Duplication events of larger DNA fragments, rather than tandem duplications, would be also consistent with the observed frequent large insertions in Sb detected by optical mapping and lack of large tandem repeat arrays (see below).

Therefore, we conclude that the increase of DNA content in the young Sb supergene is more dynamic than reported for similar (older) systems in which repeat expansions in Y chromosomes were attributed to specific satellites (Hobza et al. 2006), microsatellites (Kubat et al. 2008; Kejnovský et al. 2013) or retrotransposons (Na et al. 2014).

Since whole genome short reads have the technical limitation that at present we cannot analyse the supergene separately from the rest of the genome, we therefore cannot attribute any differences in length or numbers of specific repetitive elements to the supergene with absolute certainty. However, the measured increase of *b* repetitiveness is consistent across two different approaches and is consistent with the DNA content increase of Sb measured by optical mapping.

Large tandem repeats detected from the optical assemblies

We only detected few large tandem repeat regions within the optical assembly, mostly in unplaced optical contigs. Within the supergene, we detected two large variable repeats, specific to *S. invicta*. The first repetitive region consist of 26 repetitive units of approximately 5.50 kb in the *B* male, while the in *b* male it expanded by 3 additional repetitive units. The second repeat shows 3 repetitive units of a composite structure consisting of an approximately 3.30 kb, 3.60 kb

and 3.90 kb element in the *B* male, while the in *b* male it expanded by 4 composite units, and was found to be contracted by 2 composite units in both in *B* and *b* samples of *S. quinquecuspis* and *S. richteri* (Suppl. Fig. S10). It is important to note that only repeat arrays containing Nt.BspQI sites can be detected with the optical mapping approach.

Within raw optical mapping data we found 0.3% molecules with repeats, representing 0.1% of bases as being repetitive. Repeat unit sizes of around 5.25 kb and 12.75 kb were most abundant in the *S. invicta B* sample (Suppl. Fig. S10), as well as in all other samples (data not shown).

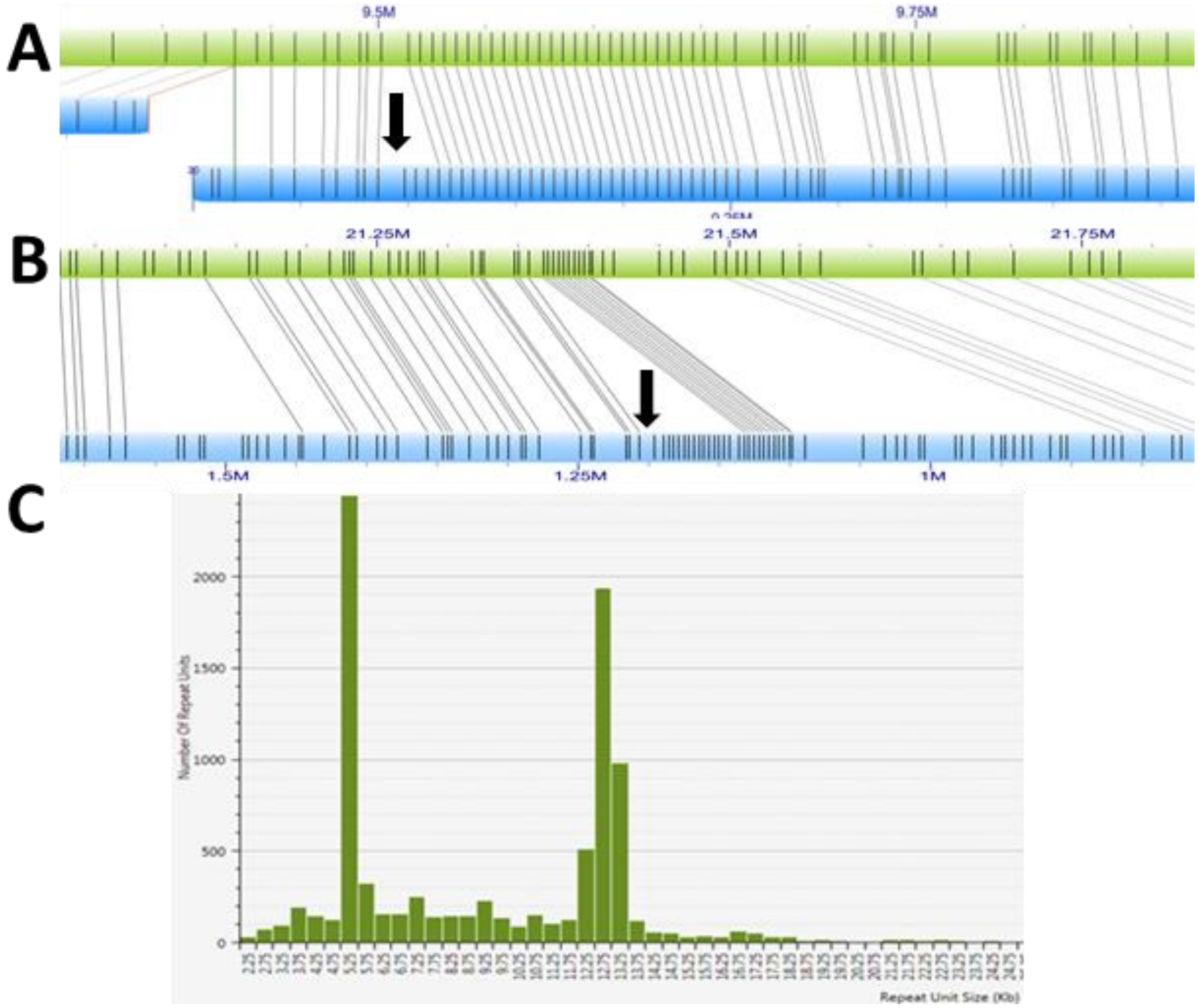

Suppl. Fig. S10. Large tandem repeat variation in two locations within the supergene and fire ant genome. **A** Expansion (+3 repeat units of 5.5 kb) in *S. invicta* Sb (compared to *S. invicta* SB). **B** Expansion (+4 repeat units of a composite repeat (3.3+3.6+3.9 kb) in *S. invicta* Sb (compared to *S. invicta* SB). **C** Repetitive fraction in *S. invicta B* raw optical molecules.

Suppl. Information 1.7. Neighbor-Joining trees for each chromosome, based on shared insertion/deletion polymorphism

To infer the phylogenetic relationship between samples based on shared indels, we performed pairwise comparisons between the optical assembly of *S. invicta B* as reference and the optical assembly from each individual as query (IrysView, BNG, v2.4). The resulting alignments (xmaps) were processed (>3kb cutoff, https://github.com/RyanONeil/structome) into BED format. We retained only regions that were covered in all six individuals, and InDels present in at least two individuals to account for differences in contiguity and mapping quality among optical assemblies and regions. We then sorted the indels by chromosome and according to the previously determined position of the optical contigs along the chromosomes. Finally, we converted this information into a pseudo-alignment by transforming indel presence to T and absence to A. This pseudo-alignment was used to infer and visualize a phylogeny based on the neighbor-joining method (Saitou and Nei 1987), Jukes-Cantor substitution model and 1000 bootstraps using the online version of MAFFT and Archeopteryx (Zmasek and Eddy 2001; Katoh and Standley 2013; Kuraku et al. 2013).

The results show a clustering of *b* and *B* individuals according to species for chromosomes 1 to 15 (Suppl. Fig. S11.1, S11.2), congruent with expectations from phylogenetic inferences based on mitochondrial sequences (see Suppl. Fig. S11.1, S11.2 and Suppl. Information 1.8). However, for chromosome 16, individuals cluster according to their supergene variant, i.e. *b* individuals of the different species cluster together, separately from the *B* individuals (Suppl. Fig. S11.1). This is congruent with expectations from phylogenetic inferences based on the *Gp9* gene (Krieger and Ross 2002) which in *S. invicta* is a marker of the supergene.

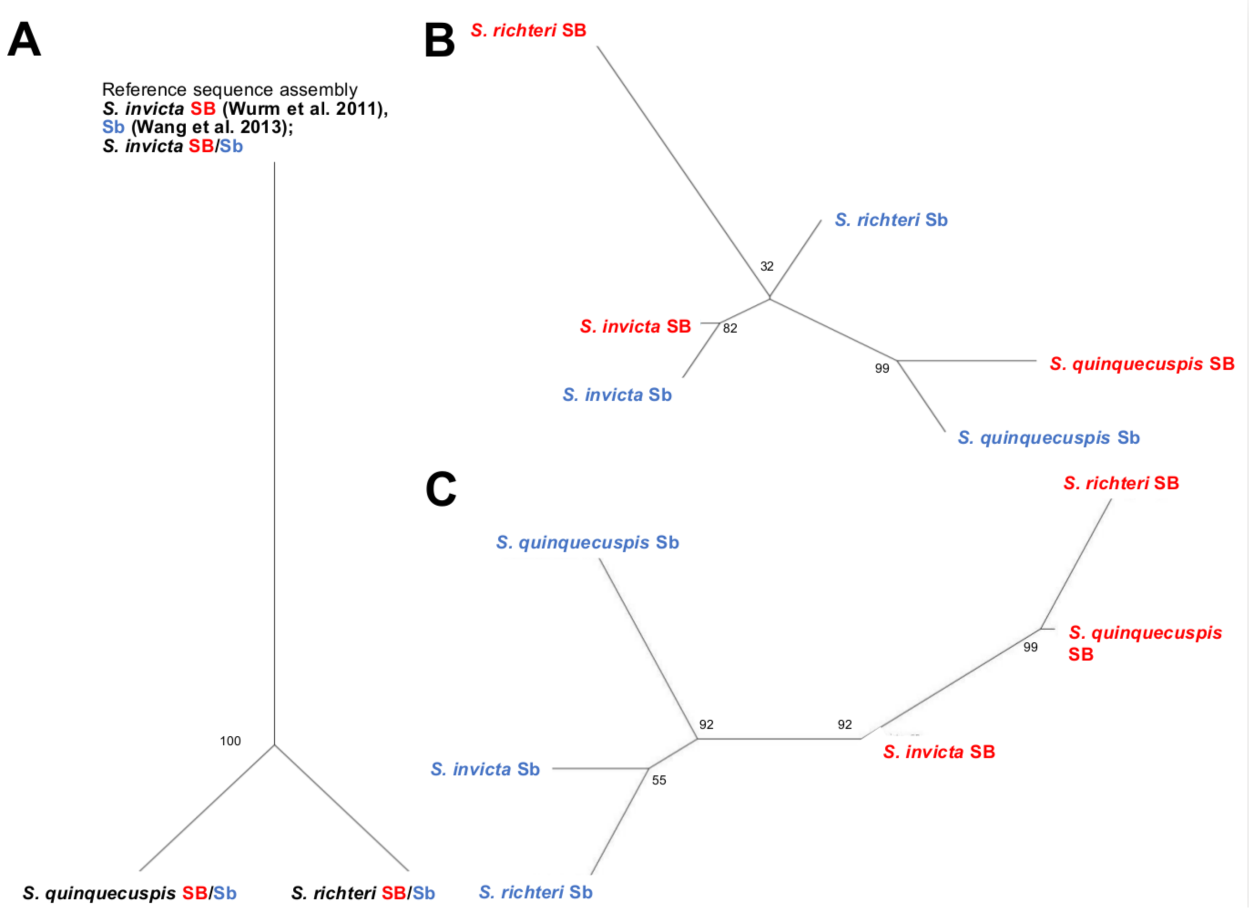

Suppl. Fig. S11.1. Summary of phylogenetic clustering of the experimental samples of *S. invicta*, *S. quinquecuspis* and *S. richteri*. **A** ML-tree (RaxML) of 777 bp mt-DNA sequences (COI) (see Suppl Information 1.8), **B** NJ-tree based of shared indels (chromosome 1), **C** NJ-tree based of shared indels (chromosomes 16).

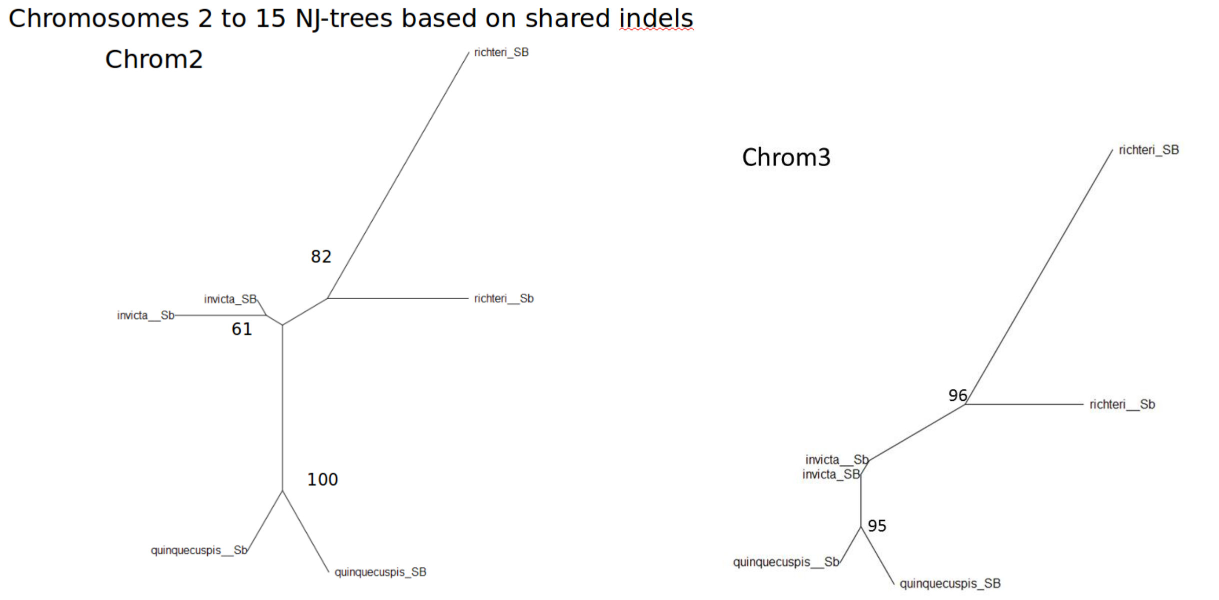

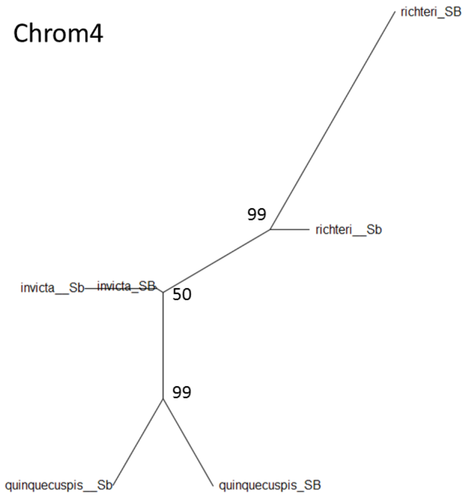
Chrom4
richteri_SB
99
richteri__Sb
invicta__Sb
invicta_SB
50
99
quinquecuspis__Sb
quinquecuspis_SB

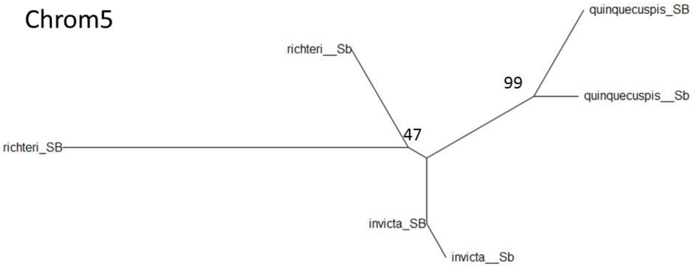
Chrom5
quinquecuspis_SB
richteri__Sb
99
quinquecuspis__Sb
47
richteri_SB
invicta_SB
invicta__Sb

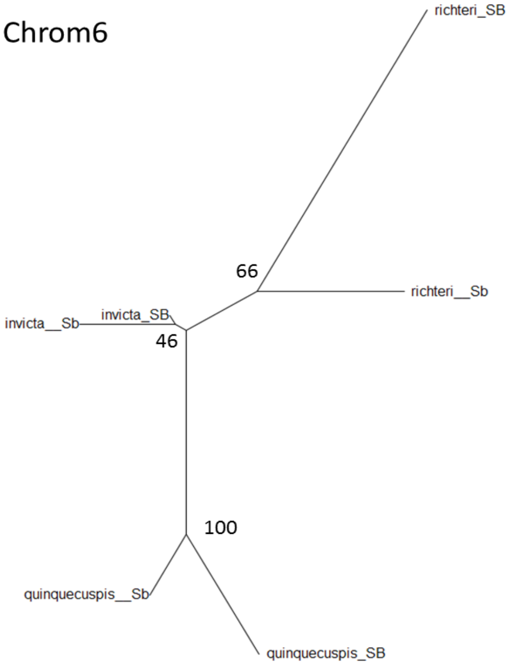
Chrom6
richteri_SB
66
richteri__Sb
invicta_SB
invicta__Sb
46
100
quinquecuspis__Sb
quinquecuspis_SB

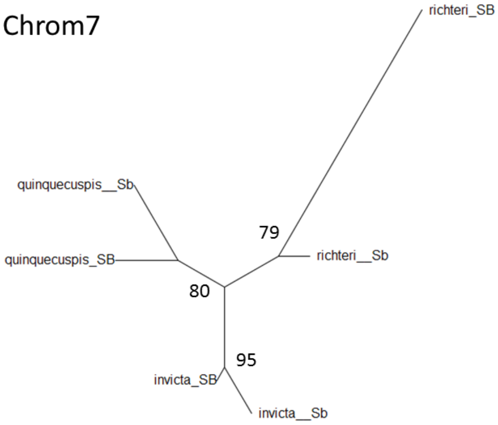
Chrom7
richteri_SB
quinquecuspis__Sb
79
quinquecuspis_SB
richteri__Sb
80
95
invicta_SB
invicta__Sb

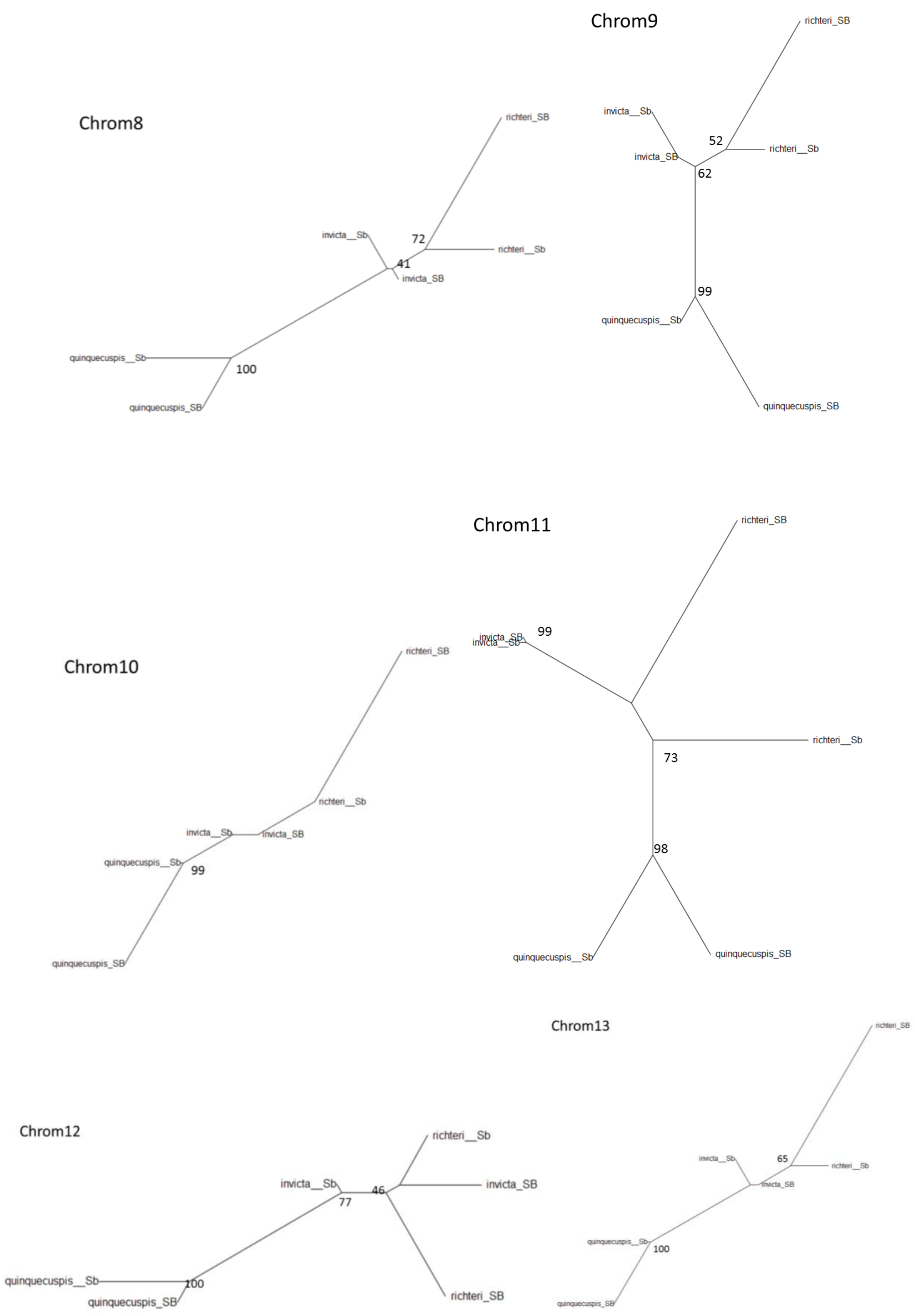
Chrom8
richteri_SB
invicta__Sb
72
richteri__Sb
41
invicta_SB
quinquecuspis__Sb
100
quinquecuspis_SB
Chrom9
richteri_SB
invicta__Sb
52
richteri__Sb
invicta_SB
62
99
quinquecuspis__Sb
quinquecuspis_SB
Chrom10
richteri_SB
richteri__Sb
invicta__Sb
invicta_SB
quinquecuspis__Sb
99
quinquecuspis_SB
Chrom11
richteri_SB
invicta_SB
invicta__Sb
99
richteri__Sb
73
98
quinquecuspis__Sb
quinquecuspis_SB
Chrom12
richteri__Sb
invicta__Sb
46
invicta_SB
77
quinquecuspis__Sb
100
quinquecuspis_SB
richteri_SB
Chrom13
richteri_SB
invicta__Sb
65
richteri__Sb
invicta_SB
quinquecuspis__Sb
100
quinquecuspis_SB

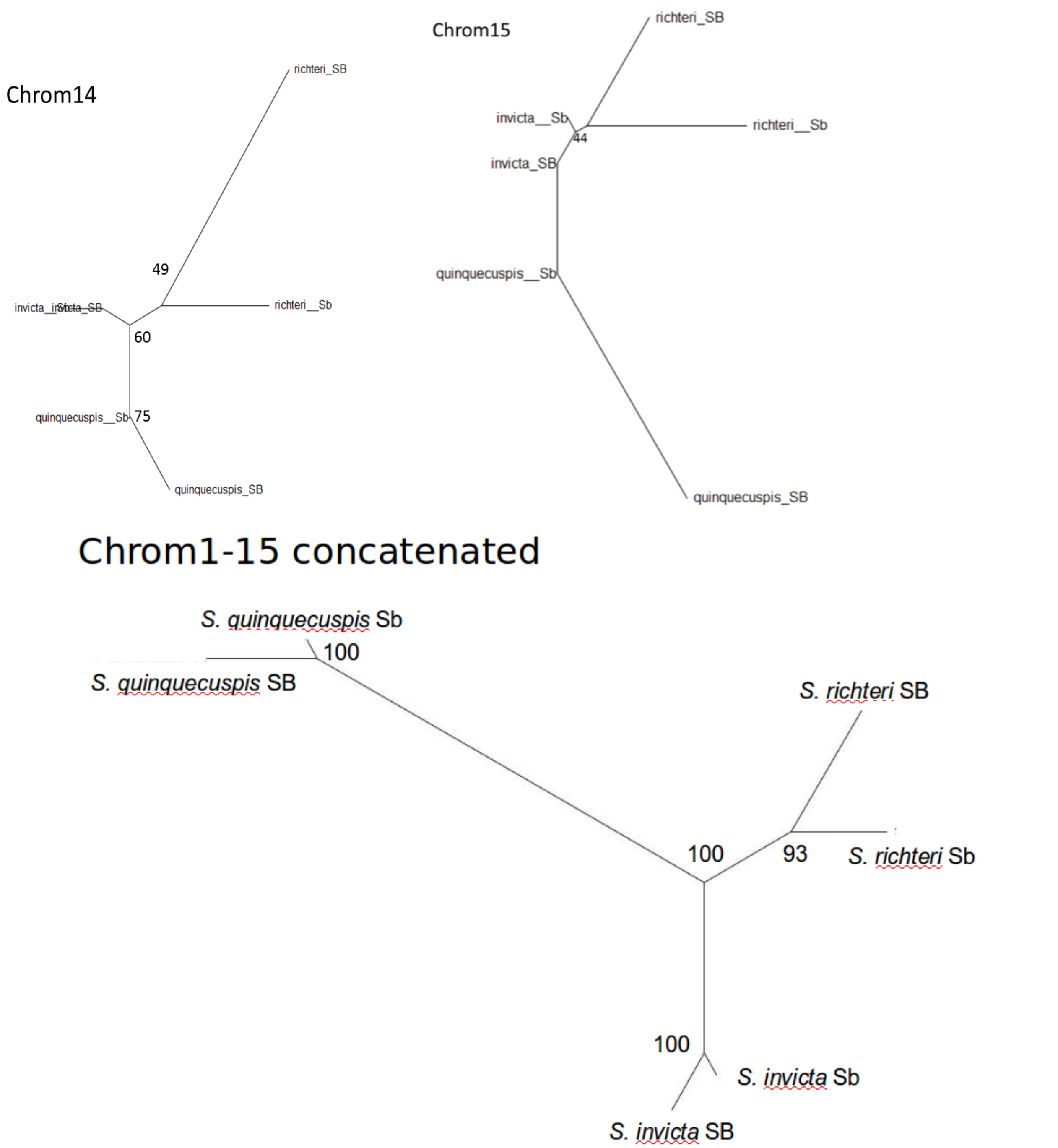

Suppl. Fig. S11.2. Phylogenetic clustering of the *b* and *B* individuals of *S. invicta*, *S. quinquecuspis* and *S. richteri*: NJ-tree based of shared indels for chromosomes 2 to 15 and the NJ-tree for the concatenated data (chromosome 1 to 15).

Suppl. Information 1.8. Phylogeny and divergence time estimation

Species identity

To establish species identity, we performed a targeted (partial) sequencing of the mitochondrially encoded cytochrome c oxidase I (COI) locus with the following PCR Primers:

SinvMT-B1F<br>5’-cattttaatcctmccyggatttgg-3’	SinvMT-B1R<br>5’- aaaatgttatatttachccaatgaatatag-3’
SinvMT-B2F<br>5’-tttccattaatctcaggataytcaataaat-3’	SinvMT-B1R<br>5’-aagatkgtgatgaagagtagaatgat-3’

Libraries were prepared from PCR fragments using NEXTflex (BIOO Scientific) and sequenced at 300 bp (paired-end) on Illumina MiSeq at Barts and the London Genome Centre (London, UK). Illumina reads were mapped with bwa-mem (Li and Durbin 2010) (v0.7.15-r1142) to the *S. invicta* mitochondrion sequence (Gotzek et al. 2010) (GenBank accession number NC_014672.1) and variants were called using freebayes (Garrison and Marth 2012) (v1.0.2-58-g054b257). Consensus FASTA sequences (GenBank accession numbers MF592128-MF592133) were extracted with vcf-consensus from vcftools (Danecek et al. 2011) (v0.1.15) and used for multiple alignment, using mafft (Katoh and Standley 2013) (v7.221, linsi) with published *Solenopsis* mitochondrion sequences (Gotzek et al. 2010) (GenBank accession numbers: *Solenopsis geminata* NC_014669.1, *S. richteri* NC_014677.1, *S. invicta* HQ215538.1, HQ215540.1, NC_014672.1) and published COI sequences (Jacobson et al. 2006; Shoemaker et al. 2006).

Phylogenetic tree construction was done using RaxML v8.0.26 with the GTR-CAT model and 1000 bootstraps (Stamatakis 2014) (Suppl. Fig. S11.1). Divergence times were estimated based with BEAST2 (Bouckaert et al. 2014) (v.2.4.7) using only the COI coding region (777 bp) of the above sequences. We used *Atta cephalotes* (Suen et al. 2011) as outgroup and used 3.91 million years as divergence time between *S. xyloni* and *S. invicta* (Moreau and Bell 2013) as prior to calibrate the tree (MCMC chain length 5 million, HKY substitution model, Calibrated Yule tree model), and tracer (Rambaut et al.) (v1.6), figtree (Rambaut and Drummond) (v1.4.3) and densitree (Bouckaert 2010) for visualization (Suppl. Fig. S12).

The topology of the resulting tree is congruent with previously inferred phylogenetic trees (Gotzek et al. 2010) (Suppl. Fig. S12). The inferred age for the divergence between *S. invicta* (HQ215540) and all other *S. invicta* or *S. richteri* and *S. quinquecuspis* was dated at 0.833 million years (ma), the split between (*S richteri* + *S.quinquecupsis*) and the remaining *S. invicta* at 0.513 ma, and the split between *S. richteri* and *S. quinquecuspis* at 0.240 ma.

Divergence time estimation based on full mitochondrial sequences

We used whole genome sequences already employed for k-mer based genome size estimations (5 pairs of *S. invicta B* and *b* male individuals, for sample details, read filtering and mapping, see Suppl. Information 1.5) to determine the sequence of the full mitochondrion for each sample from short reads. Variants were called in short reads mapped to the *S. invicta* mitochondrion using freebayes (Garrison and Marth 2012) (v1.1.0-54-g49413aa) and consensus sequences based on biallelic SNPs were extracted for each sample using bcftools (Li 2011) (v1.3.1). An alignment fasta-file of these and the *S. invicta* reference mitochondrion was created, to which previously published whole mitochondrion sequences (*S. geminata, S. richteri, S. invicta*) were added using mafft (Katoh and Standley 2013) (--add, --keeplength). Using available *S. invicta* reference mitochondrion annotations, we partitioned the alignment in BEAUti (part of the BEAST package) (codon positions 1, 2 and 3, tRNA, 12S rRNA, 16S rRNA, ATrich region, non-coding) and estimated divergence times in BEAST (Bouckaert et al. 2014) (v2.4.8) with a Calibrated Yule tree model (Heled and Drummond 2012) and in parallel using BEAGLE (Ayres et al. 2012; Ayres and Cummings 2017) (v2.1.2). We excluded genes overlapping the region found to be potentially recombinant and which therefore could have a mixed evolutionary history (nt8900-9500, genes ND6, CytB) (Gotzek et al. 2010). Results were analysed with tracer (Rambaut et al.) (v1.6), trees were annotated with treeannotator (part of the BEAST package) and displayed with figtree (Rambaut and Drummond) (v1.4.3).

As priors we used 3.91 million years as divergence time between *S. geminata* and *S. invicta* (Moreau and Bell 2013) (*S. geminata* and *S. xyloni* together are sister to all remaining taxa in this analysis), monophyly of all (*S. invicta* + *S. richteri*), HKY substitution model (gamma), strict molecular clock, and a MCMC chain length of 300 million. Results are only shown for the analysis of 3rd codon positions (the results from 1st and 2nd, and all codon positions+tRNA were similar in divergence times and identical in tree topology).

Based on 3rd codon positions from the full mitochondrion (excluding the ND6 and CytB genes) we estimated that the node containing the socially polymorphic fire ant species *S. invicta*, *S. quinquecuspis* and *S. richteri* has at least an age of 0.787 million years (0.56 - 1.03 million years) (Suppl. Fig. S12). A single *S. invicta* sample (HQ215540) appears to be potentially paraphyletic as previously pointed out (Gotzek et al. 2010). It remains unclear whether this sample belongs to a separate taxon and whether this taxon is socially polymorphic. Not considering this individual yields an age of 0.367 million years (0.25 - 0.50 million years) for the node containing the (socially polymorphic) *S. invicta* and *S. richteri* (Suppl. Fig. S12), an estimate close to the age estimate for the social chromosome (0.35 - 0.43 million years) (Wang et al. 2013).

Our results suggest similar ages of the social chromosomes and the node containing the socially polymorphic fire ant species. This supports that the social chromosome has evolved in the common ancestor. However, a paraphyletic *S. invicta* individual diverged much earlier (see above: HQ215540), raising questions about whether this individual was misidentified, and whether it represents a lineage that is also socially polymorphic. If the lineage containing the paraphyletic *S. invicta* individual also exhibits social polymorphism (*i.e.*, possessing the same social chromosome), then there would be a substantial discrepancy between the divergence

time of the socially polymorphic fire ant species and their social chromosome, hence suggesting a more complex evolutionary history, potentially by introgression of the social chromosome across hybridizing species (Jay et al. 2017).

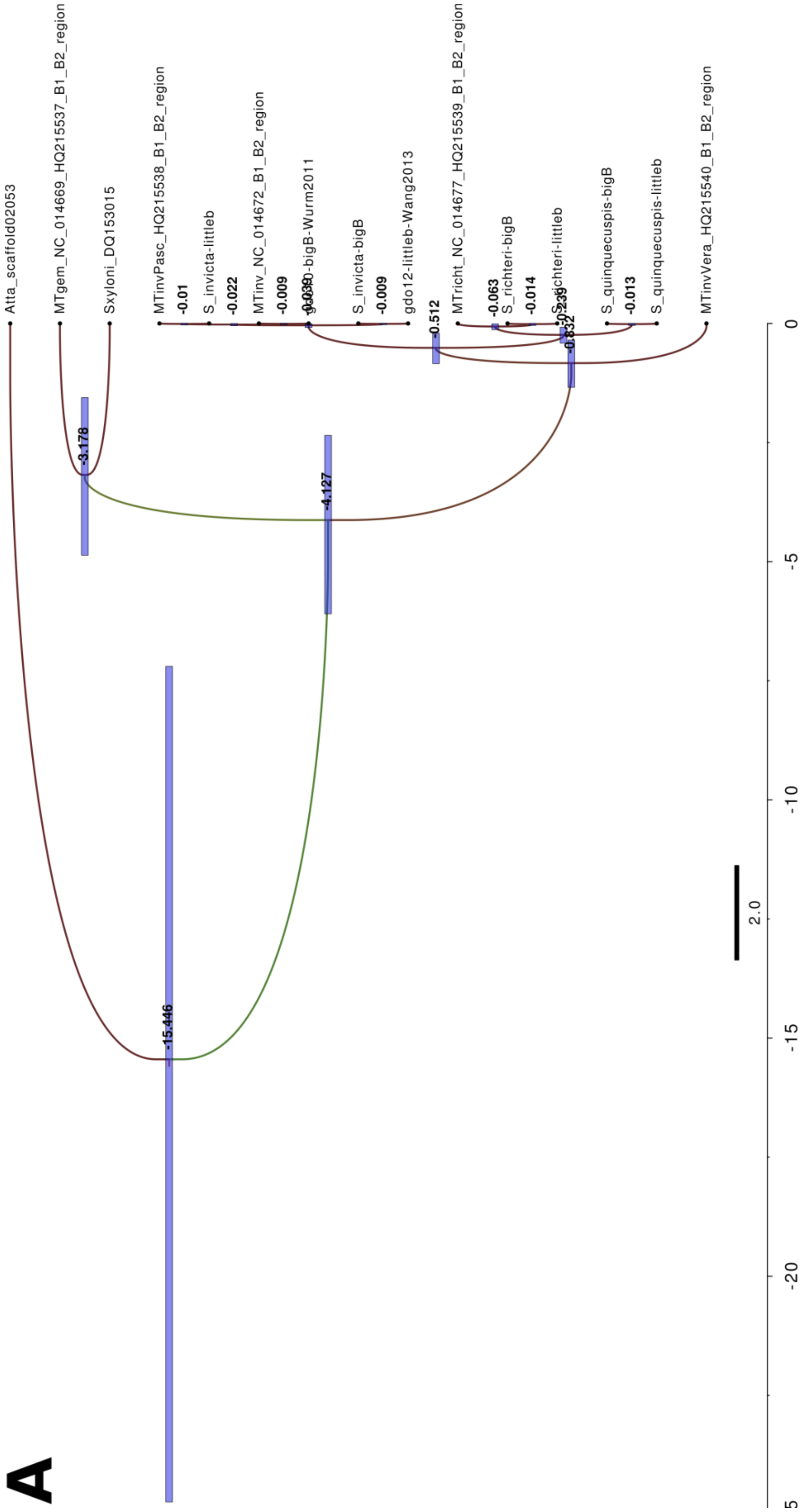

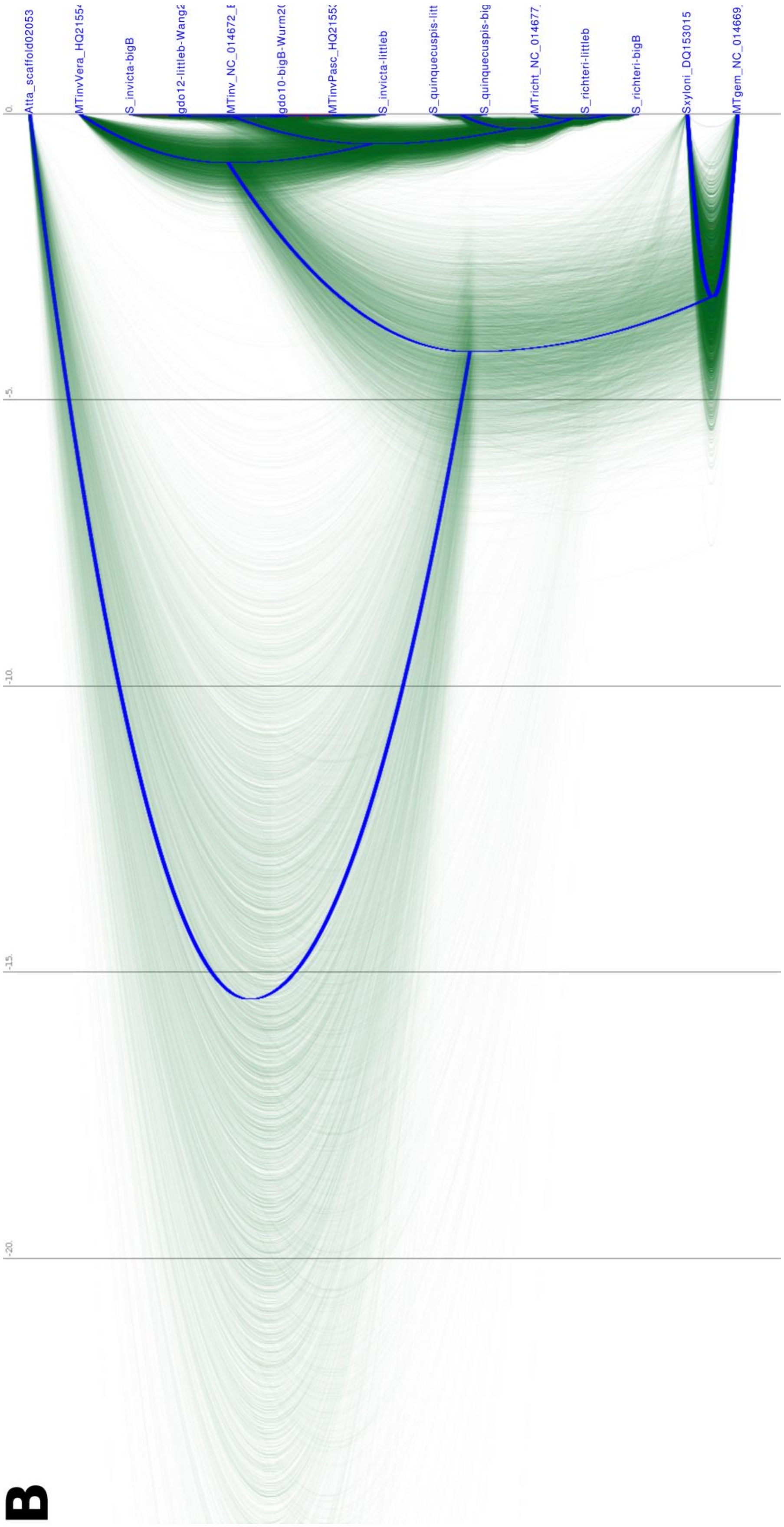
Atta_scaffold02053
MTinvVera_HQ2155
S_invicta-bigB
gdo12-littleb-Wang
MTinv_NC_014672_
gdo10-bigB-Wurm
MTinvPasc_HQ2155
S_invicta-littleb
S_quinquecuspis-litt
S_quinquecuspis-big
MTricht_NC_014677
S_richteri-littleb
S_richteri-bigB
Sxyloni_DQ153015
MTgem_NC_014669
0.
-5.
-10.
-15.
-20.
B

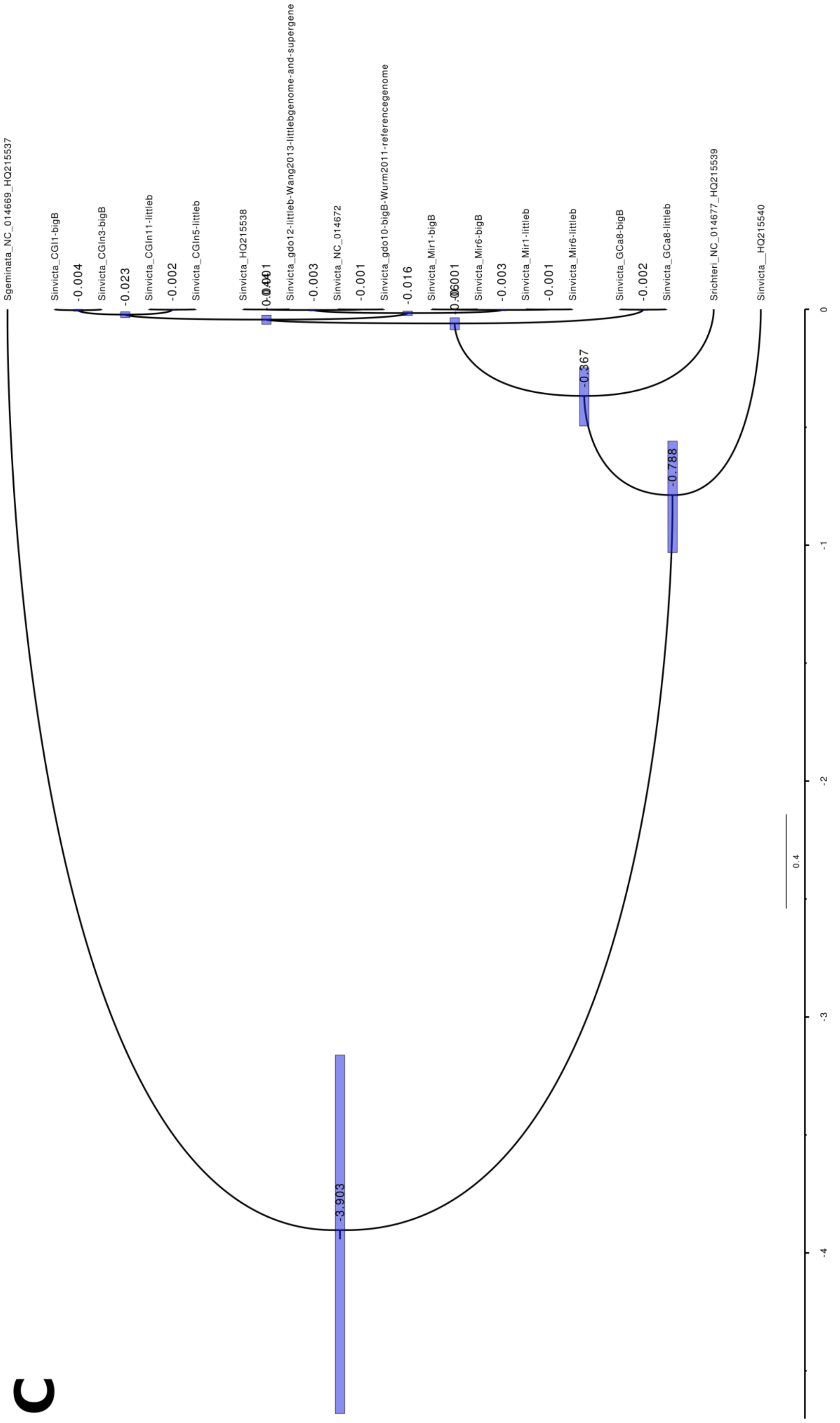
C
Sgeminata_NC_014669_HQ215537
Sinvicta_CGI1-bigB
Sinvicta_CGIn3-bigB
Sinvicta_CGIn11-littleb
Sinvicta_CGIn5-littleb
Sinvicta_HQ215538
Sinvicta_gdo12-littleb-Wang2013-littlebgenome-and-supergene
Sinvicta_NC_014672
Sinvicta_gdo10-bigB-Wurm2011-referencegenome
Sinvicta_Mir1-bigB
Sinvicta_Mir6-bigB
Sinvicta_Mir1-littleb
Sinvicta_Mir6-littleb
Sinvicta_GCa8-bigB
Sinvicta_GCa8-littleb
Srichteri_NC_014677_HQ215539
Sinvicta__HQ215540
-0.004
-0.023
-0.002
-0.003
-0.001
-0.016
-0.003
-0.001
-0.002
-0.367
-0.788
-3.903
0
-1
-2
-3
-4
0.4

Suppl. Fig. S12. A: Phylogeny (BEAST2, maximum clade credibility tree) of *Solenopsis* fire ants based on 777 bp mitochondrial COI sequence, with estimated divergence times (in million years ago [mya], shown as node labels) and 95% highest posterior density interval of divergence time (node ages). This phylogenetic tree topology is congruent with a tree produced by RaxML with the same sequences (not shown here). **B**: Phylogeny (BEAST2, densitree visualization of all trees including the consensus tree [blue]) of *Solenopsis* fire ants based on 777 bp mitochondrial COI sequence, with estimated divergence times (in million years ago [mya]) shown on the x-axis. **C**: Maximum credibility tree (BEAST2, 300 million MCMC chain length, 3rd codon position) for fire ant whole mitochondrial genomes with divergence time estimates.

Suppl. Information 1.9. Optical map alignment parameters

Suppl. Table S7. Irys View Alignment settings:

conservative alignment parameters to detect indel variation	-maxthreads 8 -res 2.9 -FP 2.0 -FN 0.30 -sf 0.3 -sd -0.15 -sr 0.08 -extend 1 -outlier 0.00001 -endoutlier 0.0001 -PVendoutlier -deltaX 8 -deltaY 8 -hashgen 5 7 2.4 1.5 0.05 5.0 1 1 1 -hash -hashdelta 50 -mres 1e-3 -hashMultiMatch 100 -insertThreads 4 -biaswt 0 -T 1e-8 -S -100 -indel -PVres 2 -rres 0.9 -MaxSE 1.8 -HSDrange 1.0 -outlierBC -AlignRes 2. -outlierExtend 12 24 -Kmax 12 -f -maxmem 128 -stdout -stderr
relaxed parameters to detect matching optical assemblies and structural rearrangements	-maxthreads 8 -res 2.9 -FP 2.0 -FN 0.10 -sf 0.04 -sd -0.25 -sr 0.09 -extend 1 -outlier 0.00001 -endoutlier 0.0001 -PVendoutlier -deltaX 8 -deltaY 8 -hashgen 5 7 2.4 1.5 0.05 5.0 1 1 1 -hash -hashdelta 50 -mres 1e-3 -hashMultiMatch 100 -insertThreads 4 -biaswt 0 -T 1e-8 -S -100 -indel -PVres 2 -rres 0.9 -MaxSE 1.8 -HSDrange 1.0 -outlierBC -AlignRes 2. -outlierExtend 12 24 -Kmax 12 -f -maxmem 128 -stdout -stderr), (-maxthreads 8 -res 2.9 -FP 0.5 -FN 0.12 -sf 0.09 -sd -0.1 -sr 0.04 -extend 1 -outlier 0.00001 -endoutlier 0.0001 -PVendoutlier -deltaX 8 -deltaY 8 -hashgen 5 7 2.4 1.5 0.05 5.0 1 1 1 -hash -hashdelta 50 -mres 1e-3 -hashMultiMatch 100 -insertThreads 4 -biaswt 0 -T 1e-6 -S -100 -indel -PVres 2 -rres 0.9 -MaxSE 1.8 -HSDrange 1.0 -outlierBC -AlignRes 2. -outlierExtend 12 24 -Kmax 12 -f -maxmem 128 -stdout -stderr
	-maxthreads 8 -res 2.9 -FP 2.0 -FN 0.30 -sf 0.3 -sd -0.15 -sr 0.08 -extend 1 -outlier 0.00001 -endoutlier 0.0001 -PVendoutlier -deltaX 8 -deltaY 8 -hashgen 5 7 2.4 1.5 0.05 5.0 1 1 1 -hash -hashdelta 50 -mres 1e-3 -hashMultiMatch 100 -insertThreads 4 -biaswt 0 -T 1e-6 -S -100 -indel -PVres 2 -rres 0.9 -MaxSE 1.8 -HSDrange 1.0 -outlierBC -AlignRes 2. -outlierExtend 12 24 -Kmax 12 -f -maxmem 128 -stdout -stderr), (-maxthreads 8 -res 2.9 -FP 1.0 -FN 0.20 -sf 0.20 -sd -0.10 -sr 0.05 -extend 1 -outlier 0.00001 -endoutlier 0.0001 -PVendoutlier -deltaX 8 -deltaY 8 -hashgen 5 7 2.4 1.5 0.05 5.0 1 1 1 -hash -hashdelta 50 -mres 1e-3 -hashMultiMatch 100 -insertThreads 4 -biaswt 0 -T 1e-6 -S -100 -indel -PVres 2 -rres 0.9 -MaxSE 1.8 -HSDrange 1.0 -outlierBC -AlignRes 2. -outlierExtend 12 24 -Kmax 12 -f -maxmem 128 -stdout -stderr
Hybrid scaffolding parameter (Irys View)	-T 1e-10 -endoutlier 1e-3 -outlier 1e-4 -extend 0 -FN 0.05 -FP 0.5 -sf 0.2 -sd 0.1 -sr 0.02 -res 0 -resSD 0.75 -mres 0 -A 5 -biaswt 0 -M 1 -Mfast 0 -deltaX 9 -deltaY 9 -RepeatMask 4 0.01 -RepeatRec 0.7 0.6 1.4 -BestRef 1 -nosplit 2 -hashgen 5 3 2.4 1.5 0.05 5.0 1 1 2 -hash -hashdelta 10 -hashmaxmem 64 -f

Suppl. Information 1.10. Simulating degenerative expansion

Methods

Our argument is that although non-recombining chromosomes accumulate deleterious mutations, they are expected to accumulate weak deleterious mutations faster than relatively stronger deleterious mutations. If deletions are more deleterious than insertions, non-recombining chromosomes are expected to increase in size.

We have developed forward simulations where we vary the fitness impact of insertions and of deletions to illustrate this argument. We simulate a population of 1000 individuals, each carrying a single non-recombining haploid chromosome. Each chromosome initially contains 4000 "sites". These represent "functional" elements such as protein-coding genes and regulatory sites. All sites are assigned an arbitrary fitness value of 10.

Each generation has three steps. First, we mutate the chromosome for each individual with insertions and deletions. The number of insertions and deletions in each individual chromosome follows a Poisson distribution with the parameter equal to the a chosen mutation rate (5×10^{-4} per site) times chromosome length. Insertions and deletions are 50 sites long and are randomly placed along the chromosome. Deletions decrease the size of the chromosome and are deleterious because they remove "functional" sites from the chromosome. Insertions increase the size of the chromosome but only affect fitness in some of our simulated conditions (see below). Second, we calculate the fitness of each individual by summing all fitness values across all of the sites on their chromosome. We determine the relative fitness of each individual by dividing their fitness by the fitness of the individual with highest fitness. In the final step, we select 1000 individuals to form the next generation by sampling them with replacement, with weighted probabilities that are equal to their relative fitnesses. We simulate for up to 1,000 generations or until one chromosome in the population is longer than 20,000 sites.

We use three additional, optional parameters in our simulations. The first is a cost for insertions, in which the alleles at the two sites directly neighbouring the insertion go from having value 10 to value 0. This is similar to how an insertion can disrupt the expression or splicing of neighboring genes. The second parameter is the addition of point mutations. These represent background degeneration of the chromosome, reducing the fitness value of affected sites by 5. If in a given lineage a single site is hit by point mutations multiple times (in different generations), the fitness of the site reduces to a minimum of 0. The number of point mutations affecting each chromosome is the result of rounding the product of point mutation rate (2.5×10^{-2}) and chromosome length. The third optional parameter is the inclusion of a class of large deletions (2,000 sites long), which happen at rare frequency in addition to the deletions and insertions sized 50.

The simulation code was written in R and is available at https://github.com/wurmlab/simulate_chromosome_length_evolution.

Results

We performed three sets of simulations, all with the same insertions and deletion rate (5×10^{-4} per site) and size (50 sites). Each set contained multiple conditions (*i.e.*, parameter settings); each condition was simulated five times.

We first tested whether having a higher cost for deletions than insertions causes an increase in size of the chromosome. In the first, "neutral" condition, representing absence of selection, all chromosomes had the same probability of being inherited in the next generation regardless of the insertions and deletions they carry. In the second condition, "deleterious deletions, neutral insertions", deletions incur a fitness cost, but insertions have no effect on fitness. Comparing these two conditions shows that the fitness cost of deletions causes an increase in the average

chromosome size in the population (Suppl. Fig. S13). We also ran a third, intermediary, condition, “deleterious deletions, deleterious insertions”, where insertions incur a fitness cost that is lower than that of deletions: the fitness value of sites immediately adjacent to the insertion is reduced by 5. In this condition, chromosome length also increases over time (Suppl. Fig. S13). Under both of these non-neutral conditions, chromosome length appears to increase indefinitely.

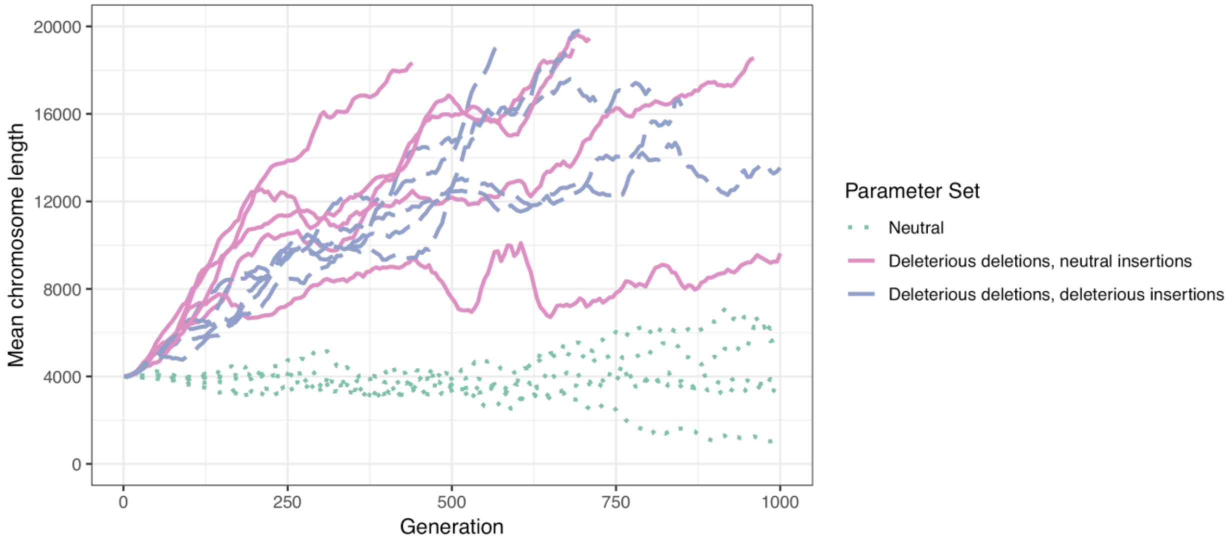

Suppl. Fig. S13: Comparison between the “Neutral”, “Deleterious deletions, neutral insertions” and “Deleterious deletions, deleterious insertions” sets of simulations, showing that inclusion of a fitness cost for deletions leads to a general increase in the average chromosome size. See text in Suppl. Information 1.10 for the parameters used in these simulations.

In Y chromosomes, different types of mutation (in addition to large insertions and deletions) cause the gradual loss of function of genes across the non-recombining region (Kaiser and Charlesworth 2010). It is intuitive that the fitness cost for deletions should be lower when there is a lower density of intact functional elements in a chromosome. To test whether background degeneration through the accumulation of point mutations can stop the growth of a non-recombining chromosome, we ran another set of simulations (“Background degeneration”). In this set, deletions incur a fitness cost, insertions have no effect on fitness, but background degeneration gradually removes fitness value from each chromosome (point mutations with a rate of 2.5×10^{-2}, where they decrease the fitness value by 5, but that have no effect if the fitness value is already 0). As in the “deleterious deletions, neutral insertions” simulation set above, the mean size of the chromosome in the population tends to increase at the start of the simulations. However, as expected if deletions become gradually less costly, this size increase eventually stops (*e.g.*, average chromosome length appears to stabilize from approximately generation 500; Suppl. Fig. S14).

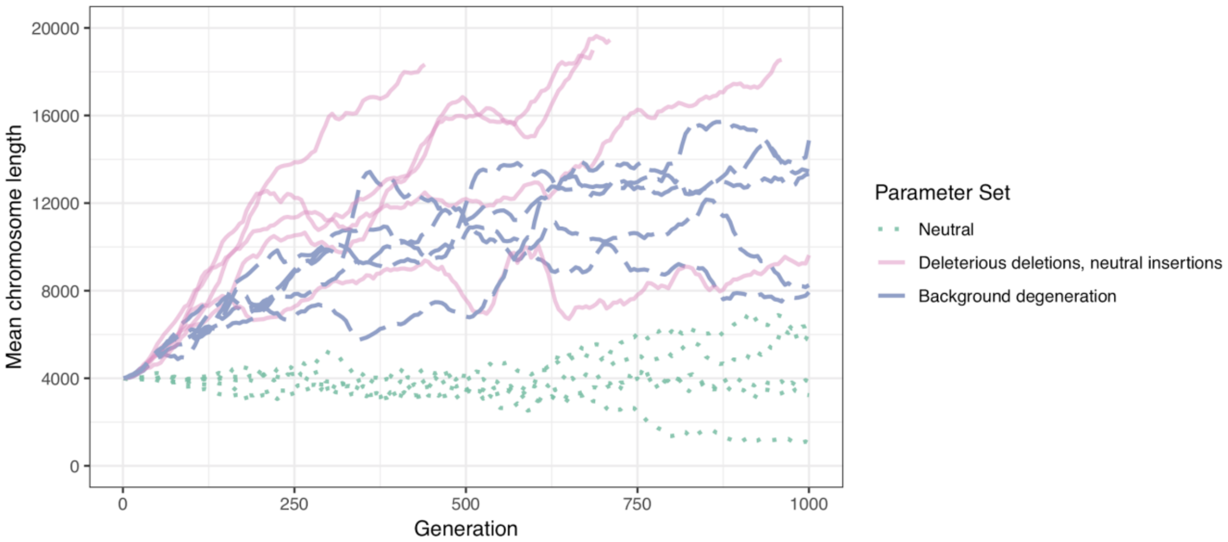

Suppl. Fig. S14: Comparison between the "Neutral", "Deleterious deletions, neutral insertions" and "Background degeneration" sets of simulations, showing that inclusion of background degeneration stops the increase of the average chromosome length.

Many ancient Y chromosomes are much smaller than their X counterparts, suggesting that there is a mechanism allowing the eventual reduction in size of a non-recombining chromosome after its initial expansion. Large deletions caused by ectopic recombination distantly positioned repeats could cause such a decreasing in the size (Devos et al. 2002; Roehl et al. 2010). Having established that an average increase in chromosome size occurs in our simulations with or without an insertion cost and with or without background selection, we tested whether simulations that include a class of large deletions can cause an eventual decrease in chromosome size. These large deletions are presumed very deleterious because they remove a very large proportion of the chromosome (2,000 sites). We performed simulations following the "deleterious deletions, neutral insertions" condition described above with an addition of this class of deletions. In the first condition, we include a relatively low mutation rate for these large deletions (1.25×10^{-6}). In the second set of simulations, we use a rate that is 20-fold higher (2.5×10^{-5}). Under both conditions, simulations showed initial increases in the average chromosome length. However, the simulations with the higher rate of large deletions subsequently decreased to below their original size (Suppl. Fig. S15).

In sum, simple forward simulations can illustrate chromosome length dynamics that occur in a non-recombining region. Although our models here are extremely simplistic, they indicate that i) if insertions are less costly than deletions, chromosome length will increase over time; ii) that background degeneration can limit this growth; and iii) that the rare occurrence of large deletions is sufficient for chromosome length to decrease once some degeneration of functional elements has occurred.

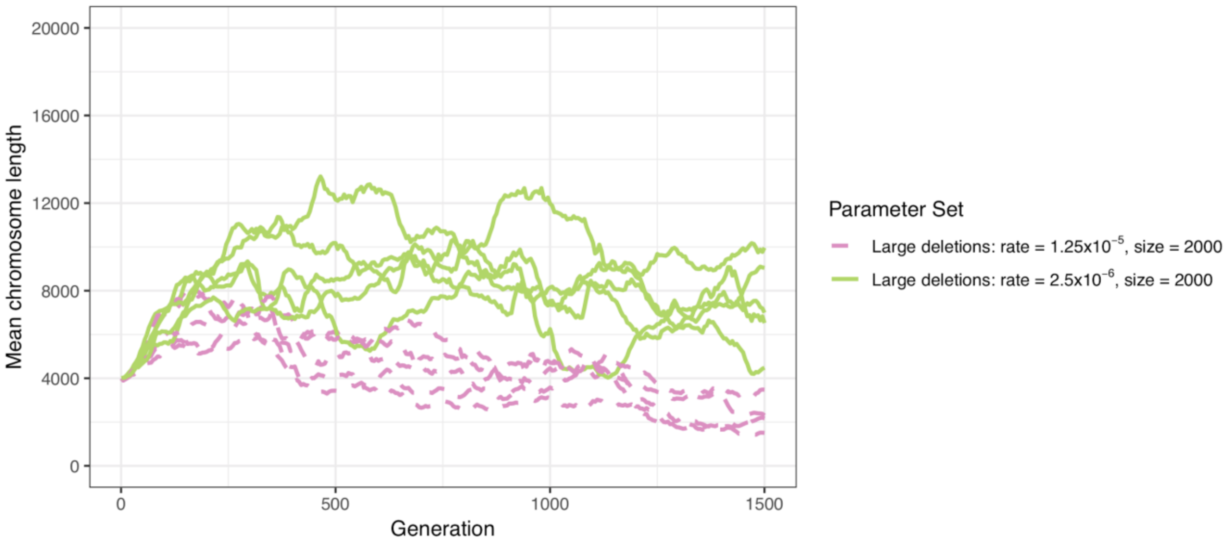

Suppl. Fig. S15: Comparison between two sets of simulations which allow large deletions in addition to small insertions and deletions. All simulations with the higher rate of large deletions (2.5×10^{-5}, in pink) had a reduction in the average size of the chromosome after generation 250. This also occurred in one of the five simulations with a lower rate (1.25×10^{-6}, in green). Note that these simulations were performed for 1,500 generations.

Other supplementary files in Supplementary Material online

Supplementary Table S8: counts and total length for each repetitive element in *B*/*b* pair of individuals (this is an Excel spreadsheet).

Supplementary Table S9: sample names and information (this is an Excel spreadsheet).

References

Ayres DL, Cummings MP. 2017. Heterogeneous Hardware Support in BEAGLE, a High-Performance Computing Library for Statistical Phylogenetics. In: 2017 46th International Conference on Parallel Processing Workshops (ICPPW). http://dx.doi.org/10.1109/icppw.2017.17

Ayres DL, Darling A, Zwickl DJ, Beerli P, Holder MT, Lewis PO, Huelsenbeck JP, Ronquist F, Swofford DL, Cummings MP, et al. 2012. BEAGLE: an application programming interface and high-performance computing library for statistical phylogenetics. Syst. Biol. 61:170–173.

Bao W, Kojima KK, Kohany O. 2015. Repbase Update, a database of repetitive elements in eukaryotic genomes. Mob. DNA 6:11.

Bouckaert R, Heled J, Kühnert D, Vaughan T, Wu C-H, Xie D, Suchard MA, Rambaut A, Drummond AJ. 2014. BEAST 2: a software platform for Bayesian evolutionary analysis. PLoS Comput. Biol. 10:e1003537.

Bouckaert RR. 2010. DensiTree: making sense of sets of phylogenetic trees. Bioinformatics 26:1372–1373.

Charif D, Lobry JR. 2007. SeqinR 1.0-2: a contributed package to the R project for statistical computing devoted to biological sequences retrieval and analysis. Structural approaches to sequence evolution:207–232.

Danecek P, Auton A, Abecasis G, Albers CA, Banks E, DePristo MA, Handsaker RE, Lunter G, Marth GT, Sherry ST, et al. 2011. The variant call format and VCFtools. Bioinformatics 27:2156–2158.

Devos KM, Brown JKM, Bennetzen JL. 2002. Genome size reduction through illegitimate recombination counteracts genome expansion in *Arabidopsis*. Genome Res. 12:1075–1079.

Garrison E, Marth G. 2012. Haplotype-based variant detection from short-read sequencing. arXiv. http://arxiv.org/abs/1207.3907

Gloor GB, Preston CR, Johnson-Schlitz DM, Nassif NA, Phillis RW, Benz WK, Robertson HM, Engels WR. 1993. Type I repressors of P element mobility. Genetics 135:81–95.

Gotzek D, Clarke J, Shoemaker D. 2010. Mitochondrial genome evolution in fire ants (Hymenoptera: Formicidae). BMC Evol. Biol. 10:300.

Goubert C, Modolo L, Vieira C, ValienteMoro C, Mavingui P, Boulesteix M. 2015. *De novo* assembly and annotation of the Asian tiger mosquito (*Aedes albopictus*) repeatome with dnaPipeTE from raw genomic reads and comparative analysis with the yellow fever mosquito (*Aedes aegypti*). Genome Biol. Evol. 7:1192–1205.

Hehir-Kwa JY, Marschall T, Kloosterman WP, Francioli LC, Baaijens JA, Dijkstra LJ, Abdellaoui A, Koval V, Thung DT, Wardenaar R, et al. 2016. A high-quality human reference panel reveals the complexity and distribution of genomic structural variants. Nat. Commun. 7:12989.

Heled J, Drummond AJ. 2012. Calibrated tree priors for relaxed phylogenetics and divergence time estimation. Syst. Biol. 61:138–149.

Hobza R, Lengerova M, Svoboda J, Kubekova H, Kejnovsky E, Vyskot B. 2006. An accumulation of tandem DNA repeats on the Y chromosome in *Silene latifolia* during early stages of sex chromosome evolution. Chromosoma 115:376–382.

Huang Y-C, Lee C-C, Kao C-Y, Chang N-C, Lin C-C, Shoemaker D, Wang J. 2016. Evolution of long centromeres in fire ants. BMC Evol. Biol. 16:189.

Hunt GJ, Page RE Jr. 1995. Linkage map of the honey bee, *Apis mellifera*, based on RAPD markers. Genetics 139:1371–1382.

Ishizaki K. 2002. Multicopy genes uniquely amplified in the Y chromosome-specific repeats of the liverwort *Marchantia polymorpha*. Nucleic Acids Res. 30:4675–4681.

Jacobson AL, Thompson DC, Murray L, Hanson SF. 2006. Establishing guidelines to improve identification of fire ants *Solenopsis xyloni* and *Solenopsis invicta*. J. Econ. Entomol. 99:313–322.

Jay P, Whibley A, Frezal L, de Cara A, Nowell RW, Mallet J, Dasmahapatra KK, Joron M. 2017. Supergene evolution triggered by the introgression of a chromosomal inversion. bioRxiv. http://dx.doi.org/10.1101/234559

Jiang H, Lei R, Ding S-W, Zhu S. 2014. Skewer: a fast and accurate adapter trimmer for next-generation sequencing paired-end reads. BMC Bioinformatics 15:182.

Kaiser VB, Charlesworth B. 2010. Muller's ratchet and the degeneration of the *Drosophila miranda* neo-Y chromosome. Genetics 185:339–348.

Katoh K, Standley DM. 2013. MAFFT multiple sequence alignment software version 7: improvements in performance and usability. Mol. Biol. Evol. 30:772–780.

Kawakatsu T, Huang S-SC, Jupe F, Sasaki E, Schmitz RJ, Urich MA, Castanon R, Nery JR, Barragan C, He Y, et al. 2016. Epigenomic Diversity in a Global Collection of *Arabidopsis thaliana* Accessions. Cell 166.

Kejnovský E, Michalovova M, Steflova P, Kejnovska I, Manzano S, Hobza R, Kubat Z, Kovarik J, Jamilena M, Vyskot B. 2013. Expansion of Microsatellites on Evolutionary Young Y Chromosome. PLoS One 8:e45519.

Kokot M, Dlugosz M, Deorowicz S. 2017. KMC 3: counting and manipulating k-mer statistics. Bioinformatics 33:2759–2761.

Kortschak RD, Tsend-Ayush E, Grützner F. 2009. Analysis of SINE and LINE repeat content of Y chromosomes in the platypus, *Ornithorhynchus anatinus*. Reprod. Fertil. Dev. 21:964–975.

Krieger MJB, Ross KG. 2002. Identification of a major gene regulating complex social behavior. Science 295:328–332.

Kubat Z, Hobza R, Vyskot B, Kejnovsky E. 2008. Microsatellite accumulation on the Y chromosome in *Silene latifolia*. Genome 51:350–356.

Kuraku S, Zmasek CM, Nishimura O, Katoh K. 2013. aLeaves facilitates on-demand exploration of metazoan gene family trees on MAFFT sequence alignment server with enhanced interactivity. Nucleic Acids Res. 41:W22–W28.

Lahn BT, Page DC. 1999. Four evolutionary strata on the human X chromosome. Science 286:964–967.

Langmead B, Salzberg SL. 2012. Fast gapped-read alignment with Bowtie 2. Nat. Methods 9:357–359.

Li H. 2011. A statistical framework for SNP calling, mutation discovery, association mapping and population genetical parameter estimation from sequencing data. Bioinformatics 27:2987–2993.

Li H, Durbin R. 2010. Fast and accurate long-read alignment with Burrows-Wheeler transform. Bioinformatics 26:589–595.

Li H, Handsaker B, Wysoker A, Fennell T, Ruan J, Homer N, Marth G, Abecasis G, Durbin R, 1000 Genome Project Data Processing Subgroup. 2009. The Sequence Alignment/Map format and SAMtools. Bioinformatics 25:2078–2079.

Li J, Heinz KM. 2000. Genome complexity and organization in the red imported fire ant *Solenopsis invicta* Buren. Genet. Res. 75:129–135.

Moreau CS, Bell CD. 2013. Testing the museum versus cradle tropical biological diversity hypothesis: phylogeny, diversification, and ancestral biogeographic range evolution of the ants. Evolution 67:2240–2257.

Na J-K, Wang J, Ming R. 2014. Accumulation of interspersed and sex-specific repeats in the non-recombining region of papaya sex chromosomes. BMC Genomics 15:335.

Pracana R, Priyam A, Levantis I, Nichols RA, Wurm Y. 2017. The fire ant social chromosome supergene variant Sb shows low diversity but high divergence from SB. Mol. Ecol. 26:2864–2879.

Quinlan AR, Hall IM. 2010. BEDTools: a flexible suite of utilities for comparing genomic features. Bioinformatics 26:841–842.

Rambaut A, Drummond A. Website. Figtree v1.4.3, http://tree.bio.ed.ac.uk/software/figtree/

Rambaut A, Suchard MA, Xie D, Drummond AJ. Tracer v1.6, http://tree.bio.ed.ac.uk/software/tracer/

R Core Team. 2013. R: A language and environment for statistical computing.

Roehl AC, Vogt J, Mussotter T, Zickler AN, Spöti H, Högel J, Chuzhanova NA, Wimmer K, Kluwe L, Mautner V-F, et al. 2010. Intrachromosomal mitotic nonallelic homologous recombination is the major molecular mechanism underlying type-2 NF1 deletions. Hum. Mutat. 31:1163–1173.

Ross KG, Krieger MJB, Shoemaker DD. 2003. Alternative genetic foundations for a key social polymorphism in fire ants. Genetics 165:1853–1867.

Saitou N, Nei M. 1987. The neighbor-joining method: a new method for reconstructing phylogenetic trees. Mol. Biol. Evol. 4:406–425.

Schwartz DC, Li X, Hernandez LI, Ramnarain SP, Huff EJ, Wang YK. 1993. Ordered restriction maps of *Saccharomyces cerevisiae* chromosomes constructed by optical mapping. Science

262:110–114.

Shi L, Guo Y, Dong C, Huddleston J, Yang H, Han X, Fu A, Li Q, Li N, Gong S, et al. 2016. Long-read sequencing and *de novo* assembly of a Chinese genome. Nat. Commun. 7:12065.

Shoemaker D, Ahrens ME, Ross KG. 2006. Molecular phylogeny of fire ants of the *Solenopsis saevissima* species-group based on mtDNA sequences. Mol. Phylogenet. Evol. 38:200–215.

Shoemaker D, Ascunce MS. 2010. A new method for distinguishing colony social forms of the fire ant, *Solenopsis invicta*. J. Insect Sci. 10:73.

Stamatakis A. 2014. RAxML version 8: a tool for phylogenetic analysis and post-analysis of large phylogenies. Bioinformatics 30:1312–1313.

Stevison LS, Hoehn KB, Noor MAF. 2011. Effects of inversions on within- and between-species recombination and divergence. Genome Biol. Evol. 3:830–841.

Suen G, Teiling C, Li L, Holt C, Abouheif E, Bornberg-Bauer E, Bouffard P, Caldera EJ, Cash E, Cavanaugh A, et al. 2011. The genome sequence of the leaf-cutter ant *Atta cephalotes* reveals insights into its obligate symbiotic lifestyle. PLoS Genet. 7:e1002007.

Sun H, Ding J, Piednoël M, Schneeberger K. 2018. findGSE: estimating genome size variation within human and Arabidopsis using k-mer frequencies. Bioinformatics 34:550–557.

Tarasov A, Vilella AJ, Cuppen E, Nijman IJ, Prins P. 2015. Sambamba: fast processing of NGS alignment formats. Bioinformatics 31:2032–2034.

Treangen TJ, Salzberg SL. 2011. Repetitive DNA and next-generation sequencing: computational challenges and solutions. Nat. Rev. Genet. 13:36–46.

Wang J, Wurm Y, Nipitwattanaphon M, Riba-Grognuz O, Huang Y-C, Shoemaker D, Keller L. 2013. A Y-like social chromosome causes alternative colony organization in fire ants. Nature 493:664–668.

Wurm Y, Wang J, Riba-Grognuz O, Corona M, Nygaard S, Hunt BG, Ingram KK, Falquet L, Nipitwattanaphon M, Gotzek D, et al. 2011. The genome of the fire ant *Solenopsis invicta*. Proc. Natl. Acad. Sci. U. S. A. 108:5679–5684.

Zmasek CM, Eddy SR. 2001. ATV: display and manipulation of annotated phylogenetic trees. Bioinformatics 17:383–384.
